# Dietary vitamin K intakes, chronic obstructive pulmonary disease, adult asthma, and lung function: a prospective cohort study in the UK Biobank

**DOI:** 10.1016/j.ajcnut.2026.101324

**Published:** 2026-04-21

**Authors:** Chengfeng Li, Pratik Pokharel, Marc Sim, Kevin Murray, Catherine P Bondonno, Benjamin H Parmenter, Liezhou Zhong, Montana Dupuy, Howraman Meteran, Jette Jakobsen, Allan Linneberg, Tilman Kühn, Aedín Cassidy, Nicola P Bondonno

**Affiliations:** 1 Nutrition and Health Innovation Research Institute, School of Medical and Health Sciences, Edith Cowan University, Joondalup, Western Australia, Australia; 2 Danish Cancer Institute, Copenhagen, Denmark; 3 School of Population and Global Health, The University of Western Australia, Perth, Western Australia, Australia; 4 Medical School, Royal Perth Hospital, The University of Western Australia, Perth, Western Australia, Australia; 5 Institute of Agriculture, The University of Western Australia, Perth, Western Australia, Australia; 6 Department of Respiratory Medicine, Copenhagen University Hospital-Hvidovre, Copenhagen, Denmark; 7 Department of Public Health, Section for Environment, Occupation, and Health, Danish Ramazzini Centre, Aarhus University, Aarhus, Denmark; 8 Research Group for Bioactives-Analysis and Application, Technical University of Denmark, Kongens Lyngby, Denmark; 9 Center for Clinical Research and Prevention, Copenhagen University Hospital-Bispebjerg and Frederiksberg, Frederiksberg, Denmark; 10 Department of Clinical Medicine, Faculty of Health and Medical Sciences, University of Copenhagen, Copenhagen, Denmark; 11 Co-Centre for Sustainable Food Systems, The Institute for Global Food Security, Queen’s University Belfast, Northern Ireland, United Kingdom; 12 Department of Nutritional, Food and Consumer Sciences, Fulda University of Applied Sciences, Fulda, Germany; 13 Center for Public Health, Medical University of Vienna, Vienna, Austria; 14 Department of Nutritional Sciences, University of Vienna, Vienna, Austria

**Keywords:** vitamin K, COPD, asthma, lung function, prospective cohort

## Abstract

**Background:**

Vitamin K–dependent proteins are important for maintaining lung structure and function, yet few studies have examined dietary vitamin K intake in relation to chronic respiratory disease.

**Objectives:**

This study aimed to investigate the associations between dietary intakes of vitamin K_1_ and vitamin K_2_ and the incidence of chronic obstructive pulmonary disease (COPD), asthma, and lung function.

**Methods:**

We analyzed data from 179,062 UK Biobank participants without COPD or asthma. Associations between dietary vitamin K_1_ and K_2_ intakes, estimated using the Oxford WebQ 24-h recall, and incident COPD and asthma, identified through hospital, death, and primary care records, were examined using Cox proportional hazards models. In cross-sectional analyses, associations of vitamin K intake with forced expiratory volume in 1 s (FEV_1_), forced vital capacity (FVC), and the FEV_1_/FVC ratio were assessed using splines within multiple regression. Stratified analyses were performed by sex, smoking status, and occupation.

**Results:**

Over 10.5-y follow-up, higher vitamin K_1_ intakes were associated with lower COPD rates, with inverse associations reaching a plateau above ∼250 *μ*g/d [HR_quintile (Q)5 compared with Q1_: 0.84; 95% confidence interval (CI): 0.75, 0.94], whereas no association was observed for vitamin K_2_. No associations were observed between vitamin K_1_ or vitamin K_2_ intakes and asthma. Higher vitamin K_1_ intakes (Q5 compared with Q1) were associated with better lung function (FVC: 44 mL; 95% CI: 35, 53 mL and FEV_1_: 32 mL; 95% CI: 25, 40 mL), whereas vitamin K_2_ showed weaker and nonlinear associations. Stronger associations between vitamin K_1_ and lung function were evident in smokers and participants with high-risk occupations.

**Conclusions:**

Higher dietary vitamin K_1_ intake was associated with better lung function and a lower rate of COPD. As vitamin K_1_ is abundant in green leafy vegetables (e.g., ∼1 serving of kale, ∼1½–2 cups), higher consumption of these foods within a healthy diet may be associated with favorable respiratory health.

## Introduction

Chronic respiratory diseases, including chronic obstructive pulmonary disease (COPD) and asthma, are among the most prevalent noncommunicable diseases worldwide and were the third leading cause of death in 2019, accounting for 4.0 million deaths and affecting 454.6 million individuals globally [1]. Although asthma and COPD can be managed with available treatments, neither condition is curable, underscoring the need for effective prevention strategies. The etiology of chronic respiratory diseases is complex and multifactorial, involving genetic, environmental, and lifestyle determinants. Among these, emerging evidence indicates that diet may play an important role in the development and progression of COPD [[2], [3], [4]] and asthma [[5], [6], [7]].

The K vitamins are a group of structurally similar compounds with shared physiological functions, primarily categorized into 2 forms. Phylloquinone (PK or vitamin K_1_) is predominantly found in green leafy vegetables, including collard greens, turnip greens, spinach, kale, broccoli, cabbage, Brussels sprouts, and lettuce [8,9]. Menaquinones (MKs, collectively known as vitamin K_2_) are mainly found in animal-based and fermented foods, with dairy products, eggs, and meat being key dietary sources [10]. These 2 forms differ in their dietary sources, metabolism, and tissue distribution. Vitamin K_1_ is preferentially retained in the liver and primarily supports hepatic functions, including blood coagulation [8], whereas vitamin K_2_ generally has a longer half-life and is more readily distributed to extrahepatic tissues where it contributes to processes such as bone metabolism and vascular health [11]. Beyond these established roles, emerging evidence suggests that vitamin K may also influence lung health [12]. Vitamin K is thought to benefit lung health by activating matrix Gla protein (MGP), a vitamin K–dependent protein that inhibits the calcification and degradation of elastin [13,14]. This suggests that vitamin K could play a protective role in developing lung diseases such as COPD and asthma. However, to date, only a limited number of studies have examined the association between vitamin K and the risk of COPD and asthma, most of which were cross-sectional and conducted in relatively small samples [[15], [16], [17]]. Although some of these studies reported associations between vitamin K status and COPD and provided limited support for a role in lung function, the overall findings have been mixed. Until recently, the lack of comprehensive dietary vitamin K intake databases has made it difficult to distinguish between the roles of vitamin K_1_ and vitamin K_2_ in respiratory health [17].

To address these gaps, the primary aim of this study was to investigate the associations between dietary intakes of vitamin K_1_ and vitamin K_2_ and the incidence of COPD and asthma. Secondary objectives were to examine the relationships between vitamin K intake and lung function parameters, including forced expiratory volume in 1 s (FEV_1_), forced vital capacity (FVC), and the FEV_1_/FVC ratio, and to explore whether these associations differed across subgroups defined by factors hypothesized to influence lung disease risk (namely, sex, smoking status, and occupation).

## Methods

### Study population

The UK Biobank is a large, population-based, prospective cohort with >500,000 participants aged 40‒69 y at recruitment between 2006 and 2010 [18]. The study included 22 assessment centers spread across England, Scotland, and Wales. Participants were recruited from National Health Service patient registers and contacted if they were located within a reasonable distance from an assessment center. At baseline, blood, urine, and saliva samples were collected, alongside detailed information on lifestyle and health, with ongoing linkage to electronic health records [19]. For the present analyses, study-specific exclusion criteria were applied to derive the analytic samples. For prospective analyses, we excluded participants with a prior diagnosis of COPD or asthma before their last 24-h dietary recall (*n* = 26,869), those using warfarin at baseline (*n* = 1585), and individuals with missing data for outcomes or covariates that only had ≤5% missing data (*n* = 1218), yielding a final sample of 179,062 participants. For cross-sectional analyses, we further excluded participants with missing spirometry data (*n* = 52,159) and those who had used an inhaler or smoked within an hour before the test (*n* = 2702), yielding a final sample of 152,727 participants (Supplementary Figure 1). All participants provided written informed consent, and ethical approval was obtained from the Northwest Multicentre Research Ethics Committee. This study was conducted and reported in accordance with the STROBE guideline.

### Dietary assessment

Dietary intake was assessed using the Oxford WebQ, a self-administered, web-based 24-h dietary questionnaire developed for use in large population studies such as UK Biobank [20]. The questionnaire captures intake of ≤206 foods and 32 drinks consumed during the previous 24 h and was administered either at baseline or during repeat assessments 2–3 y after recruitment [21]. Participants selected foods and beverages from predefined items, and intake amounts were estimated using standard portion sizes embedded within the questionnaire and linked nutrient calculation algorithms. Thus, dietary estimates were based on reported consumption over the preceding 24 h rather than on habitual frequency alone. Validation studies in the UK Biobank have demonstrated that the Oxford WebQ provides reasonably reliable estimates of dietary intake when compared with other dietary assessment approaches, including food frequency questionnaires and biomarker-based validation studies [[22], [23], [24]].

### Calculation of vitamin K intake

An overview of the food composition databases sourced to calculate PK and MK is presented in Supplementary Figure 2. Estimates of PK and MK content for each food item were primarily obtained from the UK McCance and Widdowson’s composition of foods integrated dataset [25]. When values for the exact food item were unavailable in this database, PK content was subsequently retrieved from a hierarchy of 3 additional databases [[26], [27], [28]]. Similarly, MK content was retrieved subsequently from a hierarchy of 7 additional databases [27,[29], [30], [31], [32], [33], [34]]. If no value could be assigned for a given food item across any of these sources, a value of 0 *μ*g was assigned for either PK or MK. This stepwise approach was applied to prioritize data originating from the country of interest, followed by neighboring countries, then the broader region, and finally, other regions when necessary. MKs were converted to PK equivalents using their molecular weights [31], and total vitamin K_2_ intake was calculated as the sum of individual MKs across all dietary items.

### Outcomes

#### Incident COPD and asthma

Diagnoses of COPD (International Classification of Diseases Tenth Revision, ICD-10: J44) and asthma (ICD-10: J45) were ascertained through linkage to hospital inpatient records, primary care data, and death registries, with the latter including cases where these conditions were recorded as the underlying cause of death. Only cases that occurred after the date of the last valid dietary recall were considered incident outcomes.

#### Lung function measurements

Baseline FEV_1_ and FVC were measured using a Vitalograph Pneumotrac 6800 by trained healthcare technicians and nurses at study centers as described previously [35]. Each participant initially performed 2 forced exhalations, and if the difference in FEV_1_ or FVC exceeded 5%, a third attempt was conducted. The highest values from acceptable exhalations were used for analyses.

### Covariates

Covariates were included as continuous or categorical variables as appropriate. Continuous covariates included age (years), BMI (in kg/m^2^), Townsend deprivation index, alcohol intake, physical activity, and dietary covariate (all in grams per day). Categorical covariates included sex, ethnicity, region, education, household income, smoking status (current, former, and never), smoking pack-years, number of cigarettes currently smoked daily, passive smoking, occupational risk, and number of completed plausible recalls. Detailed coding, categories, and units for all covariates are shown in Supplementary Table 1.

### Statistical analysis

Participants were followed up until the first occurrence of COPD or asthma diagnosis, death, withdrawal from the study, or the designated follow-up endpoint (31 October, 2022, for England; 31 August, 2022, for Scotland; and 31 May, 2022, for Wales). To assess associations between dietary vitamin K intake and incident COPD and asthma, we used multivariable-adjusted Cox proportional hazards models. Potential nonlinear relationships were examined using restricted cubic splines with the rcs() function from the rms R package (Frank E. Harrell). Intake variables were also categorized into quintiles (Qs), with the median intake in the lowest Q1 serving as the reference. Hazard ratios (HRs) and 95% confidence intervals (CIs) were derived from these spline models, comparing the midpoint of each Q to that of Q1, and tabulated. Additionally, associations were visualized with spline plots, displaying HRs across the range of exposure values along with corresponding 95% CI bands, with the x-axis truncated at 3 SDs above the mean. The proportional hazards assumption was evaluated using Schoenfeld residuals, and no violations were identified. To minimize potential confounding, we first constructed a directed acyclic graph to identify relevant covariates based on existing literature and a priori knowledge [2,4,36,37] **(**Supplementary Figure 3). Given the distinct dietary sources of vitamin K_1_ and vitamin K_2_, we further characterized baseline dietary intake patterns using radar plots and quantified the relative contributions of predefined food groups to total vitamin K_1_ and K_2_ intakes using food-source distribution plots (Supplementary Figures 4 and 5). These analyses were used to identify potential dietary confounders while avoiding adjustment for major dietary sources of the exposure itself. Based on these considerations, we applied 3 progressively adjusted multivariable models to evaluate the associations. Model 1 was a minimally adjusted model, including age, sex, and geographic region (the latter used as a stratification variable). Model 2 further accounted for potential sociodemographic and lifestyle confounders, including BMI, education level, Townsend deprivation index, household income, ethnicity, physical activity, combined smoking status (a composite 14-level smoking variable combining smoking status, pack-years, and cigarettes per day), passive smoking, occupation, and alcohol intake. Model 3 further accounted for dietary confounding using an exposure-specific approach reflecting the distinct food sources of vitamin K_1_ and vitamin K_2_. When vitamin K_1_ was the exposure (Model 3a), the model adjusted for intakes (grams per day) of whole grains, white meat, red and organ meat, eggs, seafood, refined grains, potatoes, nuts and seeds, sugar-sweetened beverages, tea and coffee, discretionary foods, and the number of completed plausible dietary recalls, whereas avoiding adjustment for foods that are primary sources of vitamin K_1_ (e.g., leafy vegetables). Conversely, when vitamin K_2_ was the exposure (Model 3b), the model adjusted for fruits, vegetables, whole grains, refined grains, potatoes, tea and coffee, discretionary foods, and the number of completed plausible dietary recalls. Covariates with >5% missing data were categorized into an additional “Unknown” category to retain statistical power and reduce potential selection bias associated with complete case exclusion. Continuous covariates were modeled using splines. Cross-sectional associations between exposures and spirometry outcomes were assessed using linear regression models, incorporating restricted cubic splines to capture potential nonlinear relationships and the same modeling strategy as previously described. Diagnostic plots for the spline-based linear models were inspected; no material departures from normality, homoscedasticity, or model fit were observed.

To evaluate the differences in the association between dietary vitamin K intake and the risk of incident COPD across different population subgroups, we conducted fully adjusted stratified analyses. Stratification was performed by sex (female compared with male), smoking status (never compared with ever-smokers), occupation (high-risk compared with non–high-risk with high-risk defined as occupations previously associated with increased COPD risk (e.g., mining, construction, and manufacturing) and non–high-risk as all other occupations [35]).

In additional analyses, we examined the potential interaction between vitamin K_1_ and vitamin K_2_ by cross-classifying participants according to tertiles of intake and estimating HRs for COPD, using those with low K_1_/low K_2_ as the reference group. Models were adjusted for the covariates included in Model 2. We also conducted stratified analyses by BMI categories to examine whether adiposity, given the fat-soluble nature of vitamin K and its deposition in adipose tissue, modified the association between dietary vitamin K intake and COPD risk [38]. Finally, since MK-4 and MK-9 accounted for the majority of vitamin K_2_ intake in our analytical population (∼74% and ∼11%, respectively), we conducted additional analyses examining the associations of these predominant menaquinone subtypes with COPD and asthma incidence.

To enhance the robustness of our findings, we performed several sensitivity analyses. First, we restricted the longitudinal analyses to participants who completed ≥2 dietary recalls. Second, we excluded individuals with prevalent chronic kidney disease (CKD) at baseline, given evidence that CKD is associated with subclinical vitamin K deficiency [39]. Third, we excluded participants with prevalent inflammatory bowel disease at baseline, as vitamin K deficiency is common in this population, likely due to malabsorption [40]. Fourth, to reduce potential reverse causality, we excluded individuals who were diagnosed with COPD or asthma within the first 2 y of follow-up. Fifth, we additionally adjusted for baseline air pollution exposure and history of lung diseases (yes/no) to evaluate potential residual confounding. Sixth, for the cross-sectional analyses of vitamin K and lung function outcomes, we restricted the sample to participants who completed ≥1 dietary recall during the same assessment cycle as their baseline lung function measurement, to ensure temporal alignment between exposure and outcome. Finally, as we observed a substantial number of participants without spirometry data, to assess the representativeness of the analytic sample, we compared the baseline characteristics of participants with complete spirometry data to those with missing data. All statistical analyses were performed using R version 4.4.2 (R Foundation for Statistical Computing). Statistical significance was defined as a 2-tailed *P* value ≤ 0.05 for all tests.

## Results

### Baseline characteristics

Among 179,062 participants, the median (IQR) of total vitamin K_1_ and vitamin K_2_ intakes were 104 (58, 183) *μ*g/d and 30 (28, 32) *μ*g/d, respectively. Vitamin K_1_ intake came primarily from broccoli, green leafy vegetables, and vegetable oils, whereas vitamin K_2_ intake came mainly from chicken, eggs, dairy products, and red meat (Supplementary Figure 5). A weak correlation (*r* = 0.07, *P* < 0.001) was observed between vitamin K_1_ and vitamin K_2_ intakes.

Compared with participants in the lowest vitamin K_1_ intake Q, a higher proportion of participants in the highest Q were female and had a high level of education, and they tended to be older, more physically active, and have healthier smoking profiles (Table 1). In addition, they tended to have a higher energy intake, consuming more fruits, vegetables, potatoes, wholegrains, tea and coffee, alcohol, and less refined grains (Supplementary Table 2). Compared with participants in the lowest vitamin K_2_ intake Q, a higher proportion of participants in the highest Q were male and smokers, and they tended to have a higher BMI and higher energy intake, consuming more white meat, processed meat, eggs, potatoes, discretionary foods, refined grains, and alcohol (Table 1 and Supplementary Table 2).

**TABLE 1:** Baseline characteristics of the study population

	Total population *N* = 179,062	Vitamin K_1_ intake Qs		Vitamin K_2_ intake Qs	
		Q1 *n* = 35,813	Q5 *n* = 35,812	Q1 *n* = 35,813	Q5 *n* = 35,812
Vitamin K_1_, *μ*g/d	104 (58, 183)	34 (24, 42)	287 (238, 371)	86 (45, 166)	115 (63, 203)
Vitamin K_2_, *μ*g PKeq/d^1^	30 (20, 42)	26 (16, 40)	32 (21, 45)	12 (9, 15)	56 (50, 68)
Sex (female)	98,848 (55.2)	16,920 (47.2)	22,083 (61.7)	21,602 (60.3)	17,422 (48.6)
Age (years)	60 (52, 65)	57 (50, 63)	61 (54, 66)	59 (52, 64)	59 (51, 65)
Ethnicity					
White	171,162 (95.6)	33,623 (93.9)	34,223 (95.6)	33,626 (93.9)	33,851 (94.5)
Others	7900 (4.4)	2190 (6.1)	1589 (4.4)	2187 (6.1)	1961 (5.5)
Region					
London	37,170 (20.8)	7121 (19.9)	8170 (22.8)	7822 (21.8)	7616 (21.3)
Wales	5082 (2.8)	953 (2.7)	980 (2.7)	982 (2.7)	1041 (2.9)
North-West England	22,959 (12.8)	5198 (14.5)	4017 (11.2)	4584 (12.8)	4810 (13.4)
North-East England	17,038 (9.5)	3768 (10.5)	3321 (9.3)	3353 (9.4)	3463 (9.7)
Yorkshire	28,950 (16.2)	6134 (17.1)	5727 (16.0)	5950 (16.6)	5771 (16.1)
West Midlands	15,260 (8.5)	3630 (10.1)	2828 (7.9)	3267 (9.1)	3075 (8.6)
East Midlands	10,219 (5.7)	1757 (4.9)	2170 (6.1)	1939 (5.4)	1933 (5.4)
South-East England	14,894 (8.3)	2233 (6.2)	3208 (9.0)	2684 (7.5)	2882 (8.0)
South-West England	18,579 (10.4)	3262 (9.1)	4006 (11.2)	3645 (10.2)	3452 (9.6)
Scotland	8911 (5.0)	1757 (4.9)	1385 (3.9)	1587 (4.4)	1769 (4.9)
Education					
Low	27,292 (15.2)	6693 (18.7)	5191 (14.5)	5838 (16.3)	5494 (15.3)
Medium	32,068 (17.9)	7576 (21.2)	5954 (16.6)	6311 (17.6)	6807 (19.0)
High	103,919 (58.0)	17,164 (47.9)	21,580 (60.3)	19,889 (55.5)	20,415 (57.0)
Unknown	15,783 (8.8)	4380 (12.2)	3087 (8.6)	3775 (10.5)	3096 (8.6)
Townsend deprivation index	‒2.4 (‒3.7, 0.0)	‒2.1 (‒3.6, 0.5)	‒2.4 (‒3.7, ‒0.0)	‒2.8 (‒3.7, 0.3)	‒2.2 (‒3.7, 0.2)
Income					
<£18,000	24,215 (13.5)	5504 (15.4)	5118 (14.3)	5442 (15.2)	4756 (13.3)
£18,000–£30,999	39,233 (21.9)	7731 (21.6)	8115 (22.7)	8033 (22.4)	7525 (21.0)
£31,000–£51,999	46,044 (25.7)	9164 (25.6)	8870 (24.8)	8931 (24.9)	9209 (25.7)
£52,000–£100,000	39,377 (22.0)	7505 (21.0)	7420 (20.7)	7357 (20.5)	8179 (22.8)
>£100,000	11,698 (6.5)	1999 (5.6)	2373 (6.6)	1981 (5.5)	2611 (7.3)
Unknown	18,495 (10.3)	3910 (10.9)	3916 (10.9)	4069 (11.4)	3532 (9.9)
BMI (kg/m^2^)	26.2 (23.7, 29.2)	26.9 (24.3, 30.0)	25.8 (23.4, 28.8)	25.8 (23.3, 28.7)	26.9 (24.4, 30.2)
Physical activity, MET (h/wk)	20.1 (8.7, 40.4)	17.9 (7.0, 38.5)	23.0 (10.5, 45.3)	20.7 (8.9, 41.7)	20.3 (8.6, 41.6)
Occupation					
Nonhigh risk	127,214 (71.0)	26,768 (74.7)	24,404 (68.1)	25,678 (71.7)	25,855 (72.2)
High-risk	2032 (1.1)	627 (1.8)	374 (1.0)	476 (1.3)	461 (1.3)
Unknown	49,816 (27.8)	8418 (23.5)	11,034 (30.8)	9659 (27.0)	9496 (26.5)
PM_2.5_	9.86 (9.23, 10.47)	9.93 (9.31, 10.51)	9.85 (9.19, 10.46)	9.91 (9.28, 10.52)	9.88 (9.24, 10.50)
History of lung diseases	2434 (1.4)	450 (1.3)	527 (1.5)	525 (1.5)	453 (1.3)
Smoking status					
Never smokers	102,034 (57.0)	19,697 (55.0)	20,088 (56.1)	20,947 (58.5)	19,512 (54.5)
Former smoker	63,059 (35.2)	11,989 (33.5)	13,275 (37.1)	12,065 (33.7)	13,086 (36.5)
Current smoker	13,969 (7.8)	4127 (11.5)	2449 (6.8)	2801 (7.8)	3214 (9.0)
Pack years of smoking	16 (8, 28)	19 (10, 31)	15 (8, 26)	16 (8, 27)	18 (9, 30)
Current smoking intensity, cigarettes	15 (9, 20)	15 (10, 20)	12 (7, 20)	14 (8, 20)	15 (10, 20)
Passive smoker	27,681 (15.5)	6256 (17.5)	5377 (15.0)	5491 (15.3)	6192 (17.3)

Data expressed as median (IQR) or *n* (%).

Abbreviations: MET, metabolic equivalent of task; PKeq, phylloquinone equivalent; PM_2.5_, particulate matter; Q, quintile.

1 Vitamin K_2_ intakes were converted to vitamin K_1_ equivalents based on molecular weight differences to enable appropriate summation across the different vitamin K_2_ forms.

### Associations of dietary vitamin K intake with the risk of incident COPD and asthma

**FIGURE 1:**
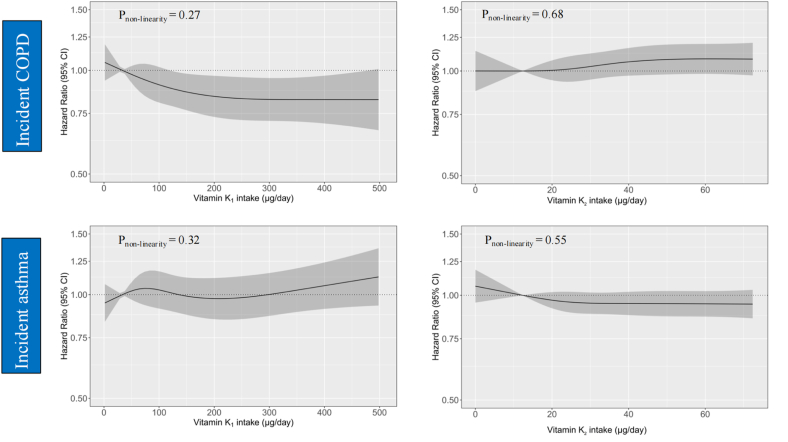
Cubic spline curves illustrating the associations between total vitamin K_1_ and vitamin K_2_ intakes and incidence of chronic obstructive pulmonary disease (COPD) and asthma among UK Biobank participants (*n* = 179,062). Hazard ratios and 95% confidence intervals (CIs) were estimated using Cox proportional hazards models adjusted for sex, age, geographic region, height, BMI, education, Townsend deprivation index, income, ethnicity, physical activity, smoking status, passive smoking, occupation, alcohol intake, and number of completed plausible dietary recalls. Models examining vitamin K_1_ intake were additionally adjusted for dietary intakes of white meat, red and organ meats, seafood, eggs, whole grains, refined grains, potatoes, nuts and seeds, sugar-sweetened beverages, tea and coffee, and discretionary foods. Models examining vitamin K_2_ intake were adjusted for intakes of fruits, vegetables, whole grains, refined grains, potatoes, tea and coffee, and discretionary foods. The reference point corresponds to the median vitamin K intake among participants in the lowest quintile (Q1).

Over a median follow-up of 10.5 y, 3135 and 4269 participants developed COPD and asthma, respectively. Higher vitamin K_1_ intakes were associated with a lower COPD rate; a nonlinear pattern was suggested visually, although the formal test for nonlinearity was not significant, with HRs reaching a plateau at intake amounts above ∼250 *μ*g/d (Figure 1). Participants in the highest Q of vitamin K_1_ intake had a 16% lower rate of COPD compared with those in the lowest Q [HR_Q5 compared with Q1_ (95% CI): 0.84 (0.75, 0.94), Model 3a]. No significant associations were observed between vitamin K_1_ intake and asthma [HR_Q5 compared with Q1_ (95% CI): 0.99 (0.91, 1.09), Model 3a] nor between vitamin K_2_ intakes and either COPD or asthma [HR_Q5 compared with Q1_ for COPD: 1.08 (0.98, 1.20); HR_Q5 compared with Q1_ asthma: 0.95 (0.87, 1.03), Model 3b] (Table 2).

**TABLE 2:** Hazard ratios for incident chronic obstructive pulmonary disease and asthma by quintiles of vitamin K intake

*N* = 179,062	Vitamin K intake Qs				
	Q1 = 35,813	Q2 = 35,813	Q3 = 35,812	Q4 = 35,812	Q5 = 35,812
Vitamin K_1_, (*μ*g/d)^1^	34 (24, 42)	66 (58, 74)	104 (93, 116)	163 (145, 183)	287 (238, 371)
Incident COPD					
Cases/*n*	817/35,813	652/35,813	575/35,812	513/35,812	578/35,812
HR (95% CI)					
Model 1	ref.	0.73 (0.69, 0.78)	0.60 (0.55, 0.65)	0.57 (0.52, 0.61)	0.58 (0.53, 0.64)
Model 2	ref.	0.95 (0.89, 1.01)	0.90 (0.83, 0.99)	0.86 (0.79, 0.93)	0.83 (0.75, 0.92)
Model 3a	ref.	0.97 (0.91, 1.04)	0.93 (0.85, 1.02)	0.88 (0.80, 0.96)	0.84 (0.75, 0.94)
Incident asthma					
Cases/*n*	914/35,813	867/35,813	815/35,812	804/35,812	869/35,812
HR (95% CI)					
Model 1	ref.	0.91 (0.86, 0.96)	0.86 (0.79, 0.93)	0.84 (0.78, 0.90)	0.86 (0.79, 0.94)
Model 2	ref.	0.98 (0.93, 1.04)	0.96 (0.89, 1.04)	0.94 (0.87, 1.02)	0.95 (0.87, 1.04)
Model 3a	ref.	1.00 (0.94, 1.07)	1.00 (0.92, 1.09)	0.98 (0.91, 1.07)	0.99 (0.91, 1.09)
Vitamin K_2_, (*μ*g PKeq/d)^1^^,^^2^	12 (9, 15)	22 (20, 24)	30 (28, 32)	39 (37, 42)	56 (50, 68)
Incident COPD					
Cases/*n*	606/35,813	562/35,813	624/35,812	634/35,812	709/35,812
HR (95% CI)					
Model 1	ref.	0.93 (0.86, 0.99)	0.95 (0.87, 1.03)	1.03 (0.94, 1.12)	1.14 (1.04, 1.25)
Model 2	ref.	1.00 (0.93, 1.07)	1.01 (0.93, 1.10)	1.04 (0.96, 1.14)	1.08 (0.98, 1.19)
Model 3b	ref.	1.01 (0.93, 1.08)	1.03 (0.94, 1.12)	1.06 (0.97, 1.16)	1.08 (0.98, 1.20)
Incident asthma					
Cases/*n*	916/35,813	835/35,813	790/35,812	880/35,812	848/35,812
HR (95% CI)					
Model 1	ref.	0.93 (0.87, 0.98)	0.92 (0.86, 0.98)	0.95 (0.88, 1.02)	0.99 (0.91, 1.07)
Model 2	ref.	0.96 (0.90, 1.01)	0.94 (0.88, 1.01)	0.95 (0.88, 1.02)	0.95 (0.88, 1.03)
Model 3b	ref.	0.96 (0.91, 1.02)	0.95 (0.89, 1.02)	0.95 (0.88, 1.02)	0.95 (0.87, 1.03)

A total of 3135 and 4269 participants were diagnosed with COPD and asthma, respectively, over a median follow-up period of 10.5 y. HRs and 95% CIs were estimated using restricted cubic spline functions within Cox proportional hazards models, with pointwise estimates comparing the midpoint of each Q to the reference midpoint of the first Q1. Model 1 adjusted for sex, age, and region; model 2 additionally adjusted for BMI, education, Townsend deprivation index, income, ethnicity, region, physical activity, smoking status, passive smoking, occupation, and alcohol; model 3a additionally adjusted for number of completed plausible recalls, white meat, red and organ meat, seafood, eggs, wholegrains, refined grains, potatoes, nut and seeds, sugar-sweetened beverages, tea and coffee, and discretionary foods; model 3b adjusted for all variables in model 2, plus fruits, vegetables, whole grains, refined grains, potatoes, tea and coffee, discretionary foods, and the number of completed plausible dietary recalls.

Abbreviations: CI, confidence interval; COPD, chronic obstructive pulmonary disease; HR, hazard ratio; PKeq, phylloquinone equivalent; Q, quintile; ref., reference.

1 Median (IQR).

2 Vitamin K_2_ intakes were converted to vitamin K_1_ equivalents based on molecular weight differences to enable appropriate summation across the different vitamin K_2_ forms.

### Cross-sectional associations of dietary vitamin K intake with lung function parameters

**FIGURE 2:**
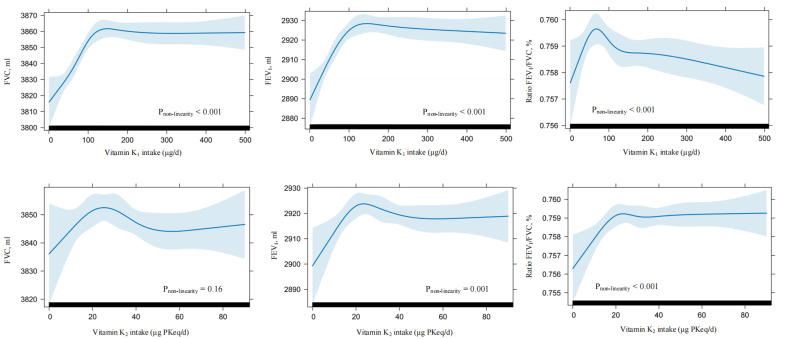
Graphical representation of the multivariable-adjusted dose–response relationships between dietary vitamin K intake and lung function parameters—forced vital capacity (FVC, mL), forced expiratory volume in 1 s (FEV_1_, mL), and the FEV_1_/FVC ratio (%)—in participants from the UK Biobank. Estimated β coefficients and 95% confidence intervals (CIs) were derived from restricted cubic spline terms in linear regression models, adjusted for sex, age, geographic region, height, BMI, education, Townsend deprivation index, income, ethnicity, physical activity, smoking status, passive smoking, occupation, alcohol intake, number of completed plausible dietary recalls, models examining vitamin K_1_ intake were additionally adjusted for dietary intakes of white meat, red and organ meats, seafood, eggs, whole grains, refined grains, potatoes, nuts and seeds, sugar-sweetened beverages, tea and coffee, and discretionary foods. Models examining vitamin K_2_ intake were adjusted for intakes of fruits, vegetables, whole grains, refined grains, potatoes, tea and coffee, and discretionary foods. Blue shaded areas indicate the 95% CIs. The rug plots along the x-axis represent individual participant observations. *P* values for nonlinearity were derived from likelihood ratio tests. PKeq, phylloquinone equivalent.

Among 152,727 participants with spirometry data, higher intakes of vitamin K_1_ were significantly associated with better lung function**.** Dose–response curves showed strong nonlinear relationships (*P*_nonlinearity_ < 0.001 for FVC and FEV_1_), with lung function improving steeply up to ∼150 *μ*g/d before reaching a plateau, whereas the FEV_1_/FVC ratio declined slightly at higher intakes (Figure 2). In fully adjusted models (Model 3a), participants with moderate to high intakes (Q4) of vitamin K_1_ intake had, on average, a 45 mL higher FVC (95% CI: 37, 53), a 37 mL higher FEV_1_ (95% CI: 30, 44) and a 0.10% better FEV_1_/FVC ratio (95% CI: 0.02, 0.18) than those with low intakes (Q1). Estimates for FVC and FEV_1_ remained similar in the highest intake category (Q5), whereas the association with the FEV_1_/FVC ratio was not significant at the highest intake amount (Table 3).

**TABLE 3:** Cross-sectional associations between vitamin K intake and lung function parameters

*N* = 152,727	Vitamin K intake Qs				
	Q1 = 30,456	Q2 = 30,546	Q3 = 30,547	Q4 = 30,547	Q5 = 30,547
Vitamin K_1_, (*μ*g/d)^1^	34 (25, 43)	67 (59, 75)	105 (94, 117)	164 (146, 184)	285 (238, 368)
FVC, mL					
Model 1	ref.	78 (71, 84)	126 (117, 135)	140 (131, 149)	139 (129, 148)
Model 2	ref.	27 (21, 33)	44 (36, 52)	49 (42, 57)	49 (40, 57)
Model 3a	ref.	24 (18, 30)	40 (32, 48)	45 (37, 53)	44 (35, 53)
FEV_1_, mL					
Model 1	ref.	62 (57, 68)	99 (92, 107)	106 (99, 113)	100 (92, 108)
Model 2	ref.	23 (18, 28)	37 (30, 44)	39 (32, 45)	34 (27, 41)
Model 3a	ref.	22 (17, 27)	35 (28, 42)	37 (30, 44)	32 (25, 40)
FEV_1_/FVC, ratio (%)					
Model 1	ref.	0.12 (0.06, 0.18)	0.15 (0.07, 0.23)	0.07 (‒0.01, 0.15)	‒0.06 (‒0.14, 0.03)
Model 2	ref.	0.09 (0.03, 0.15)	0.12 (0.04, 0.20)	0.07 (‒0.01, 0.14)	‒0.02 (‒0.11, 0.06)
Model 3a	ref.	0.11 (0.05, 0.17)	0.15 (0.06, 0.23)	0.10 (0.02, 0.18)	0.01 (‒0.08, 0.10)
Vitamin K_2_, (*μ*g PKeq/d)^1^^,^^2^	13 (9, 15)	22 (20, 24)	30 (28, 32)	39 (37, 42)	56 (50, 67)
FVC, mL					
Model 1	ref.	40 (31, 48)	48 (37, 58)	38 (26, 49)	21 (9, 34)
Model 2	ref.	17 (10, 25)	19 (10, 28)	12 (3, 22)	5 (‒6, 16)
Model 3b	ref.	20 (12, 27)	22 (13, 31)	15 (5, 25)	8 (‒3, 19)
FEV_1_, mL					
Model 1	ref.	33 (26, 40)	40 (32, 49)	33 (25, 42)	23 (13, 32)
Model 2	ref.	17 (11, 24)	20 (12, 27)	14 (6, 22)	8 (‒1, 17)
Model 3b	ref.	20 (13, 26)	23 (16, 31)	18 (9, 26)	11 (2, 20)
FEV_1_/FVC, ratio (%)					
Model 1	ref.	0.10 (0.02, 0.17)	0.14 (0.05, 0.23)	0.15 (0.06, 0.25)	0.19 (0.08, 0.29)
Model 2	ref.	0.13 (0.05, 0.20)	0.16 (0.07, 0.24)	0.14 (0.04, 0.23)	0.11 (0.01, 0.21)
Model 3b	ref.	0.14 (0.07, 0.22)	0.18 (0.10, 0.27)	0.17 (0.07, 0.26)	0.15 (0.04, 0.25)

Estimates (β) and 95% CIs were obtained from restricted cubic splines within linear regression models comparing the median exposure intakes in Q2–Q5 to the median exposure intakes in Q1. Model 1 adjusted for sex, age, and region; model 2 additionally adjusted for height, BMI, education, Townsend deprivation index, income, ethnicity, region, physical activity, smoking status, passive smoking, occupation, and alcohol; model 3a additionally adjusted for number of completed plausible recalls, white meat, red and organ meat, seafood, eggs, wholegrains, refined grains, potatoes, nut and seeds, sugar-sweetened beverages, tea and coffee, and discretionary foods. Model 3b adjusted for all variables in Model 2, plus fruits, vegetables, whole grains, refined grains, potatoes, tea and coffee, discretionary foods, and the number of completed plausible dietary recalls.

However, 52 159 participants had missing data for predicted FEV_1_ as their spirometry did not meet European Respiratory Society/American Thoracic Society criteria.

Abbreviations: CI, confidence interval; FEV1, forced expiratory volume in 1 s; FVC, forced vital capacity; PKeq, phylloquinone equivalent; Q, quintile; ref., reference.

1 Median (IQR).

2 Vitamin K_2_ intakes were converted to vitamin K_1_ equivalents based on molecular weight differences to enable appropriate summation across the different vitamin K_2_ forms.

Vitamin K_2_ intake also showed positive associations with lung function, though the effect estimates were smaller and nonlinear patterns were less pronounced (Figure 2). In fully adjusted models (Model 3b), participants with moderate vitamin K_2_ intakes (Q3) had, on average, a 22 mL higher FVC (95% CI: 13, 31) and a 23 mL higher FEV_1_ (95% CI: 16, 31) than those with low intakes (Q1), with estimates declining for higher intakes (Q4 and Q5).

### Stratified analyses

**FIGURE 3:**
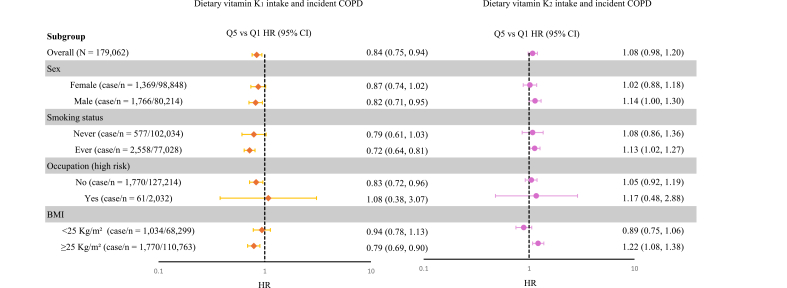
Forest plots illustrate stratified associations between dietary vitamin K intake and chronic obstructive pulmonary disease (COPD) incidence. The x-axis represents the hazard ratios (HR) on a logarithmic scale. Analyses were stratified by key demographic and clinical subgroups, including age, sex, smoking status, occupation, and the presence of chronic conditions: chronic kidney disease and inflammatory bowel disease. HRs and 95% confidence intervals (CIs) were estimated using Cox proportional hazards models with vitamin K_1_ intake modeled as restricted cubic splines. Associations compare the median intake in the highest quintile (Q)5 to that in the lowest quintile (Q1, reference). Models examining vitamin K_1_ intake were additionally adjusted for dietary intakes of white meat, red and organ meats, seafood, eggs, whole grains, refined grains, potatoes, nuts and seeds, sugar-sweetened beverages, tea and coffee, and discretionary foods. Models examining vitamin K_2_ intake were adjusted for intakes of fruits, vegetables, whole grains, refined grains, potatoes, tea and coffee, and discretionary foods.

Stratified analyses showed that the inverse association between higher vitamin K_1_ intake and COPD incidence was generally consistent across subgroups, although several subgroup-specific estimates were not statistically significant (Figure 3). Higher vitamin K_1_ intake was also associated with better FVC and FEV_1_ in most subgroups, with stronger effect estimates observed in ever-smokers than never-smokers [β_Q5 compared with Q1_ (95% CI): FVC: 69 mL (56, 82)mL compared with 33 mL (21, 45) mL; FEV_1_: 60 mL (49, 72) mL compared with 23 mL (14, 33) mL], and participants in high-risk occupations compared with non–high-risk occupations [β_Q5 compared with Q1_ (95% CI): FVC: 116 mL (22, 211) mL compared with 47 mL (36, 57) mL; FEV_1_: 74 mL (‒7, 156) mL compared with 37 mL (28, 46) mL] although the latter FEV_1_ estimate was not statistically significant (Supplementary Figure 6). By contrast, vitamin K_2_ intake showed no clear or consistent pattern across stratified analyses. For incident COPD, a statistically significant positive association was observed only among smokers [HR_Q5 compared with Q1_ (95% CI): 1.13 (1.02, 1.27)] but not never-smokers [HR_Q5 compared with Q1_ (95% CI): 1.08 (0.86, 1.36)], whereas the estimate in males [HR_Q5 compared with Q1_ (95% CI): 1.14 (1.00, 1.30)] and females [HR_Q5 compared with Q1_ (95% CI): 1.02 (0.88, 1.18)] were similar in direction and magnitude (Figure 3). For lung function, moderate vitamin K_2_ intake was associated with more favorable FVC, FEV_1_, and FEV_1_/FVC estimates in all subgroups investigated except for FEV_1_/FVC ratio among those with a high-risk occupation. (Supplementary Figure 7).

### Additional analyses

When we explored the potential interplay between vitamin K_1_ and vitamin K_2_ by cross-classification, we observed that higher vitamin K_1_ intake exhibited a trend toward lower COPD risk among participants with low vitamin K_2_ intake, but this association was not evident among those with high vitamin K_2_ intake (Supplementary Table 3). In BMI-stratified analysis, among participants with BMI ≥25, higher vitamin K_1_ intake was associated with a lower rate of incident COPD [HR_Q5 compared with Q1_ (95% CI): 0.79 (0.69, 0.90)] and a better lung function (Figure 3 and Supplementary Figures 6 and 7). For MK subtype analysis, neither MK-4 nor MK-9, the 2 predominant menaquinone subtypes in this cohort, was significantly associated with incident COPD or asthma (Supplementary Tables 4 and 5).

### Sensitivity analyses

In sensitivity analyses, the associations between vitamin K intakes and both COPD risk and lung function outcomes remained consistent with the primary findings. When restricting the analysis to participants who completed ≥2 dietary recalls, similar results were obtained. Specifically, higher vitamin K_1_ intake showed a trend toward lower risk of COPD (HR 0.90; 95% CI: 0.77, 1.04), although the association was not statistically significant (Supplementary Table 6). The inverse association between vitamin K_1_ intake and COPD risk also persisted after excluding individuals with prevalent CKD or inflammatory bowel disease at baseline (Supplementary Tables 7 and 8). Excluding incident COPD cases occurring within the first 2 y of follow-up to minimize reverse causality did not materially alter the findings (Supplementary Table 9). Additional adjustment for baseline air pollution exposure and history of lung diseases did not modify the findings (Supplementary Table 10). In the cross-sectional analyses, restricting the sample to participants whose dietary recall coincided with the baseline lung function assessment produced effect estimates for FVC, FEV_1_, and FEV_1_/FVC that were comparable with those observed in the primary analysis (Supplementary Table 11). In participants who had a missing value of the spirometry test, we observed a similar covariate pattern, including critical variables like smoking traits, which are key covariates in the study. (Supplementary Table 12).

## Discussion

### Main findings

In this cohort of over 179,000 United Kingdom adults, we found that higher habitual intakes of vitamin K_1_ were associated with a lower rate of COPD and better lung function. Specifically, participants in the highest Q of vitamin K_1_ intake had a 16% lower rate of COPD compared with those in the lowest Q, and higher vitamin K_1_ intakes were associated with a better FVC and FEV_1_ but not FEV_1_/FVC ratio. By contrast, vitamin K_2_ intake was not associated with COPD and demonstrated weaker and less consistent relationships with lung function. Additionally, neither vitamin K_1_ nor vitamin K_2_ was associated with incident adult asthma. Taken together, these findings suggest a potentially important role for vitamin K_1_ in the primary prevention of COPD and the preservation of lung function.

### Comparison with previous studies

Our findings are consistent with and extend the existing literature on vitamin K and respiratory health. To date, most comparable studies have been cross-sectional. For example, in a cross-sectional study of 4092 individuals, a low vitamin K status, indicated by elevated amounts of the vitamin K–dependent protein desphospho-uncarboxylated MGP, was associated with a 98 mL lower FEV_1_ (95% CI: 54, 141 mL), a 136 mL lower FVC (95% CI: 85, 187 mL) and significantly higher odds of COPD (odds ratio: 2.24; 95% CI: 1.53, 3.27) [16]. In our study, we observed that higher dietary vitamin K_1_ intakes, which should lead to a higher vitamin K status, were associated with better lung function and lower COPD rates. Similarly, a cross-sectional analysis of 17,681 US adults showed that meeting the recommended daily vitamin K intake was associated with 39% lower odds of emphysema (odds ratio: 0.61; 95% CI: 0.40, 0.92) after adjusting for smoking and other confounders [17]. Another study comparing 98 cases of COPD and 986 controls also reported that low vitamin K status was associated with a higher odd of COPD [15]. Of note, our study substantially expands this evidence base, providing prospective data from a large-scale cohort demonstrating an inverse association between higher dietary vitamin K_1_ intake and COPD risk. Although the associations between vitamin K_1_ intake and lung function were significant, the absolute differences observed were modest. Higher vitamin K_1_ intake was associated with ∼30–45 mL higher FEV_1_ and FVC, comparable to ∼1 y to 2 y of age-related lung function decline in middle-aged adults (∼20–30 mL FEV_1_ per year). In contrast, smoking-related deficits are substantially larger. Thus, although small at the individual level, modest shifts in lung function may still be meaningful at the population level. It is noteworthy that the vitamin K_1_ intake amounts observed in the higher Qs of our study exceed current dietary recommendations and average intakes reported in population-based dietary surveys. Although estimated adequate intakes are typically around 1 *μ*g/kg body weight per day, surveys across Europe and North America generally report mean intakes ranging from ∼70‒200 *μ*g/d [8,41]. Therefore, the intake amounts observed in the upper Qs of the present study are higher than typical population averages. However, these findings should not be interpreted as evidence that intakes above current dietary recommendations are required for respiratory health. Rather, higher vitamin K_1_ intakes in this cohort likely reflect dietary patterns characterized by greater consumption of green leafy vegetables and other nutrient-rich plant foods. Consequently, the observed associations may capture broader dietary behaviors associated with healthier lifestyles rather than a specific requirement for elevated vitamin K_1_ intake. Although the null association observed for vitamin K_2_ intake in the present study may reflect differences in bioavailability, tissue distribution, or potential physiological function between PK (vitamin K_1_) and MKs (vitamin K_2_) [42], it is more plausibly explained by differences in their dietary sources and associated dietary patterns. Vitamin K_1_ is primarily obtained from green leafy vegetables such as broccoli, spinach, and lettuce, whereas vitamin K_2_ is largely derived from animal products, e.g., chicken, eggs, and cheese [42]. Diets rich in green leafy vegetables are typically higher in polyphenols, fiber, and other micronutrients with antioxidant and anti-inflammatory properties, which may synergistically promote respiratory health [43]. In contrast, major sources of vitamin K_2_ often co-occur with foods linked to adverse health outcomes, such as processed and red meats. This heterogeneity of food sources, where beneficial nutrients coexist with potentially harmful dietary components, may have attenuated the observed associations between vitamin K_2_ intake and lung health in this study. Likewise, we observed no associations between dietary vitamin K_1_ or vitamin K_2_ intake and the incidence of adult-onset asthma. This finding aligns with prior cohort studies and systematic reviews that have generally reported null associations between diet quality or antioxidant-rich dietary patterns and asthma risk [44]. The absence of association may reflect fundamental pathophysiological differences between asthma and COPD. Whereas oxidative stress and tissue degradation are central to COPD and may be influenced by nutrient-related mechanisms, adult-onset asthma is characterized by complex immune dysregulation and airway remodeling that are less amenable to modification by dietary vitamin K intake in adulthood [45].

### Potential biological mechanism

Several biological mechanisms may explain the protective association between vitamin K_1_ and COPD. Vitamin K functions as an essential cofactor for the carboxylation of MGP, a potent inhibitor of soft-tissue calcification that is abundantly expressed in lung tissue [14]. In the lungs, the active (carboxylated) form of MGP preserves elastic fiber integrity by preventing aberrant calcium deposition. In contrast, vitamin K deficiency leads to the accumulation of inactive (uncarboxylated) MGP, allowing calcification and degradation of elastic fibers, processes implicated in the pathogenesis of emphysema and COPD [14]. Consistent with this mechanism, COPD patients with low vitamin K status (reflected by high amounts of uncarboxylated MGP) have demonstrated accelerated elastin degradation and more severe emphysema [46]. Importantly, COPD is increasingly recognized as a heterogeneous condition comprising distinct pathological phenotypes, including emphysema-predominant and airway-predominant disease. The vitamin K–dependent mechanisms described above, particularly those involving MGP-mediated preservation of elastic fibers and inhibition of tissue calcification, may therefore be relevant to emphysema-dominant COPD characterized by alveolar destruction and elastin degradation. However, our study could not differentiate COPD phenotypes. Future studies incorporating imaging-based phenotyping or molecular markers will be needed to determine whether vitamin K_1_ differentially influences specific COPD subtypes. Notably, the dose–response analysis revealed a plateau of the association between vitamin K_1_ intake and COPD incidence beyond ∼250 *μ*g/d (Q5), which may suggest a threshold effect whereby the biological benefits of vitamin K_1_, such as sufficient MGP carboxylation and elastic fiber preservation, are largely achieved at this level, with additional intake yielding diminishing returns. However, caution is warranted when comparing these intake values to established dietary reference values, as self-reported dietary estimates are not validated as absolute intake measures and should not be interpreted as such. The association between higher vitamin K_1_ intake and better lung function, characterized by higher FVC and FEV_1_ but only a modestly higher FEV_1_/FVC ratio, suggests that vitamin K may primarily help preserve lung volume and compliance rather than markedly improving airflow obstruction [47]. This pattern is consistent with a general improvement in lung capacity or preservation of lung tissue integrity. Notably, the association was strongest at moderate vitamin K_1_ intake amounts, with little additional benefit at the highest intake category. This plateau may reflect a threshold effect, whereby vitamin K–dependent pathways relevant to lung structure and function reach functional sufficiency at moderate intake amounts.

The inverse association between higher vitamin K_1_ intake and COPD incidence was generally consistent across population subgroups. However, stronger associations between higher vitamin K_1_ intake and better lung function were observed between smokers and participants in high-risk occupations—groups characterized by greater exposure to inhaled toxins and oxidative stress. Notably, stratified analyses should be considered exploratory given that subgroups with smaller case numbers, such as those in high-risk occupations, are subject to reduced statistical power and wide CIs, and findings from these analyses should not be over-interpreted. Although these findings are broadly consistent with previous studies suggesting stronger protective associations of antioxidant-rich dietary patterns among smokers [[48], [49], [50], [51]], they do not imply that dietary factors can offset the harmful effects of tobacco exposure. Indeed, smoking cessation remains the primary strategy for preventing COPD. Previous evidence on antioxidant supplementation in smokers has produced inconsistent and, at times, paradoxical results, with some trials even reporting increased risks of smoking-related cancers following high-dose supplementation of antioxidant vitamins and minerals [52]. These observations underscore that dietary modification should be viewed as a complementary, rather than substitutive, strategy for disease prevention. In this context, higher consumption of vitamin K_1_-rich foods such as green leafy vegetables may be part of dietary patterns supportive of lung health, but such measures should not detract from the critical importance of eliminating tobacco exposure. For vitamin K_2_, no clear or consistent patterns were observed across stratified analyses.

Exploratory cross-classification of vitamin K_1_ and vitamin K_2_ intakes suggested a trend toward lower COPD risk with higher K_1_ intake among those with low vitamin K_2_ intake, which may reflect differences in overall dietary patterns between those with high and low K_2_ intake rather than a direct biological interaction between the 2 vitamins. However, HRs across joint Q categories did not follow a consistent or monotonic pattern, and this finding should be interpreted with caution and considered hypothesis-generating rather than conclusive. In BMI-stratified analyses, the inverse association between higher vitamin K_1_ intake and COPD risk appeared stronger among participants with higher BMI. Although previous studies suggest that adipose tissue may sequester vitamin K and reduce its availability for other physiological functions, our findings indicate that the relationship may be more complex [38]. Hence, these results raise the possibility that BMI may modify vitamin K metabolism or requirements. However, these findings are exploratory, and intervention studies are needed to clarify this relationship.

### Strengths and limitations

This study has several limitations that warrant discussion. First, residual confounding by lifestyle and dietary factors cannot be entirely excluded, despite rigorous adjustment for a wide range of covariates. Although we adjusted for numerous dietary and lifestyle factors and explored dietary patterns using radar plots and food-source analyses, vitamin K_1_ may partly act as a marker of a broader plant-rich dietary pattern. Therefore, the observed associations should not be interpreted as definitive evidence of a causal effect of vitamin K_1_ alone. Second, misclassification of vitamin K intake is possible, as estimates were derived from self-reported, short-term dietary recalls. Although the Oxford WebQ used in this study has demonstrated acceptable validity for ranking individuals’ dietary intakes when compared with repeated 24-h recalls among UK Biobank participants [20,21], the specific vitamin K_1_ and vitamin K_2_ intake estimates derived for the present study have not been formally validated. Therefore, within-person variability and nutrient-specific measurement error may still have introduced exposure misclassification. In our data, the intraclass correlation coefficient across repeated recalls was 0.247 for vitamin K_1_ and 0.199 for vitamin K_2_. Such levels of within-person variability may lead to regression dilution bias and attenuation of associations, particularly for vitamin K_2_. Third, we did not have biochemical measures of vitamin K status, and therefore our results reflect dietary intake rather than functional status or absorption efficiency. Although intake-based measures are relevant for dietary guidance, discordance between intake and status can arise in circumstances such as fat malabsorption, use of vitamin K-antagonist medications, or age-related changes in absorption efficiency, and future studies incorporating biomarkers such as desphospho-uncarboxylated MGP may better capture functional vitamin K status. Fourth, although our findings are most generalizable to middle-aged and older adults in the United Kingdom, replication in other populations and settings will be important to confirm external validity. However, the large, well-characterized cohort and prospective design strengthened the reliability of the observed associations. Finally, vitamin K supplementation was not included in our analysis. However, reported use was very uncommon in this cohort (<0.1% of participants) and is therefore unlikely to have materially influenced our findings. Accordingly, our exposure estimates primarily reflect dietary vitamin K intake. Our study has several strengths. First, the large sample size and prospective design enabled robust estimation of long-term associations between vitamin K intake and incident respiratory outcomes, thereby reducing the likelihood of reverse causation. Second, to date, as no comprehensive food database of vitamin K intake was available, we systematically combined existing databases and prioritized those most relevant to the study population. This approach allowed us to generate more accurate and context-specific estimates of dietary vitamin K intake. However, although the systematic combination of existing databases and prioritization of those most relevant to the study population aimed to maximize accuracy, the database approach inevitably introduces some uncertainty in absolute intake estimates, for example, database values may not fully capture cooking-related changes in vitamin K content, regional and seasonal variation in the vitamin K content of foods, or differences between the specific food products consumed and those represented in the database. Third, by integrating both longitudinal and cross-sectional analyses, we were able to assess vitamin K’s role in respiratory disease and lung function from complementary perspectives, strengthening the overall conclusions. Finally, the consistency of findings across multiple stratified and sensitivity analyses supports the robustness and credibility of our results.

In conclusion, from a public health perspective, increasing green leafy vegetables, e.g., kale, by 1 serve (∼1½–2 cups) a day could help achieve higher vitamin K_1_ intakes within the context of a healthy diet. However, future clinical trials and mechanistic studies are warranted to determine whether increasing dietary vitamin K_1_ or pharmaceutical vitamin K supplementation can causally reduce COPD risk or improve lung function.

## Author contributions

The authors’ responsibilities were as follows – CL, PP, MS, KM, CPB, NPB: contributed to the study concept and design; CL, PP, LZ, MD: calculated vitamin K intakes from processed food frequency questionnaire data; CL, BHP, PP: conducted the data analysis under the guidance of KM and NPB; TK, AC: processed, calculated and refined all the data from the UK Biobank databases; CL, NPB: drafted the manuscript; CL, PP, MS, KM, CPB, BHP, LZ, MD, HM, JJ, AL, TK, AC, NPB: critically reviewed the manuscript; and all authors: read and approved the final manuscript.

## Data availability

Data from the UK Biobank are available to all researchers upon application (apply for the use of the UK Biobank dataset at http://ukbiobank.ac.uk/register-apply/). A description of all variables used (including UK Biobank identification number and any transformations or categorizations performed) is included in the Supplemental Material. This research was conducted using UK Biobank-funded and sourced data (application 64426).

## Declaration of Generative AI and AI-assisted technologies in the writing process

During the preparation of this work, the authors used ChatGPT in order to assist with English language optimization. After using this tool/service, the authors reviewed and edited the content as needed and take full responsibility for the content of the publication.

## Funding

This study was supported by a grant from Independent Research Fund Denmark (3101-00054B). PP is supported by a research grant from the Danish Diabetes and Endocrine Academy which is funded by the Novo Nordisk Foundation (grant number: NNF22SA0079901). The salary of CPB is funded by a National Health and Medical Research Council Ideas Grant (grant number: APP2030071) and the Western Australian Future Health Research and Innovation Fund (grant number: WANMA/EL2023-24/2), an initiative of the Western Australian State Government. The salary of LZ is supported by an Emerging Leader Fellowship from the Western Australian Future Health Research and Innovation Fund (identification number: WANMA/EL2022/8), an initiative of the Western Australian State Government, and a National Health and Medical Research Council of Australia Ideas Grant (identification number: 2028286). BHP was supported by a Postdoctoral Fellowship (110456-2025) from the National Heart Foundation of Australia and a Priming Grant from the Raine Medical Research Foundation (RPG027-2025). This research was supported by Research Ireland, Northern Ireland’s Department of Agriculture, Environment and Rural Affairs, and UK Research and Innovation via the International Science Partnerships Fund (grant number: 22/CC/11147) at the Co-Centre for Sustainable Food Systems. This study was also supported by resources provided by the Pawsey Supercomputing Research Centre’s Setonix Supercomputer (https://doi.org/10.48569/18sb-8s43), with funding from the Australian Government and the Government of Western Australia.

## Conflict of interest

BHP has consulted for MaxBiocare, a company engaged in micronutrient research and related commercial activities. All other authors report no conflicts of interest.
