# Supplementary material for: Dietary vitamin K intakes, chronic obstructive pulmonary disease, adult asthma, and lung function: a prospective cohort study in the UK Biobank
Source: Am J Clin Nutr. 2026 Apr 21;123(6):101324. doi: 10.1016/j.ajcnut.2026.101324 (PMC13269342; doi:10.1016/j.ajcnut.2026.101324)
Supplement: Multimedia component 1 [file mmc1.docx]

**Dietary vitamin K intakes, chronic obstructive pulmonary disease, adult asthma, and lung function: a prospective cohort study in the UK Biobank**

*Li et al.,*

**Supplementary Material**


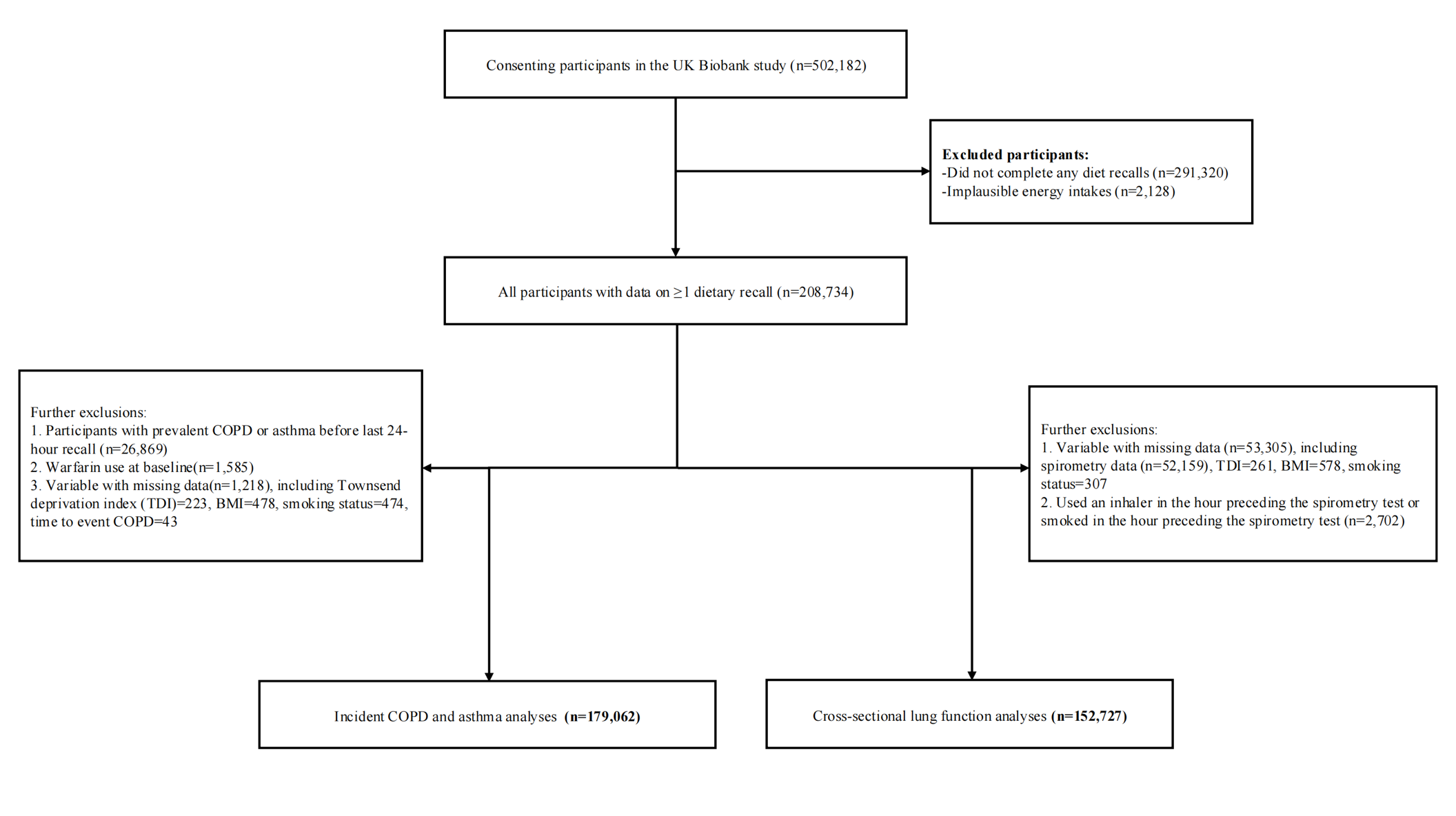
**Supplementary Figure 1.** Flowchart of the participants selection in the UK Biobank.

**Supplementary Figure 2.** Flowchart for the hierarchy of international databases used for the calculations of dietary PK (four databases) and MK (eight databases), respectively

References

1. GOV.UK. Composition of foods integrated dataset (CoFID). 2021.
2. Bolton-Smith C, Price RJ, Fenton ST, et al. Compilation of a provisional UK database for the phylloquinone (vitamin K1) content of foods. *Br J Nutr* 2000;83:389–99.
3. US Department of Agriculture. FoodData Central.
4. Presse N, Potvin S, Bertrand B, et al. Phylloquinone content of herbs, spices and seasonings. J Food Compos Anal 2015;41:15–20.
5. Schurgers LJ, Vermeer C. Determination of phylloquinone and menaquinones in food. Effect of food matrix on circulating vitamin K concentrations. *Haemostasis* 2000;30:298–307.
6. Manoury E, Jourdon K, Boyaval P, et al. Quantitative measurement of vitamin K2 (menaquinones) in various fermented dairy products using a reliable high-performance liquid chromatography method. *J Dairy Sci* 2013;96:1335–46.
7. Jensen MB, Langwagen M, Christensen T, et al. Vitamin K content in food and dietary intake among the Danes. *Food Chem* 2025;464:141651.
8. DTU Food Institute. Frida - Database with food data.
9. RIVM. Dutch food composition database (NEVO).
10. Palmer CR, Koch H, Shinde S, et al. Development of a vitamin K database for commercially available food in Australia. *Front Nutr* 2021;8:753059.


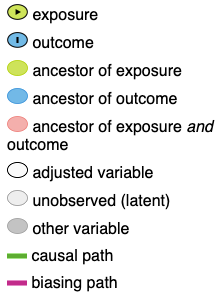

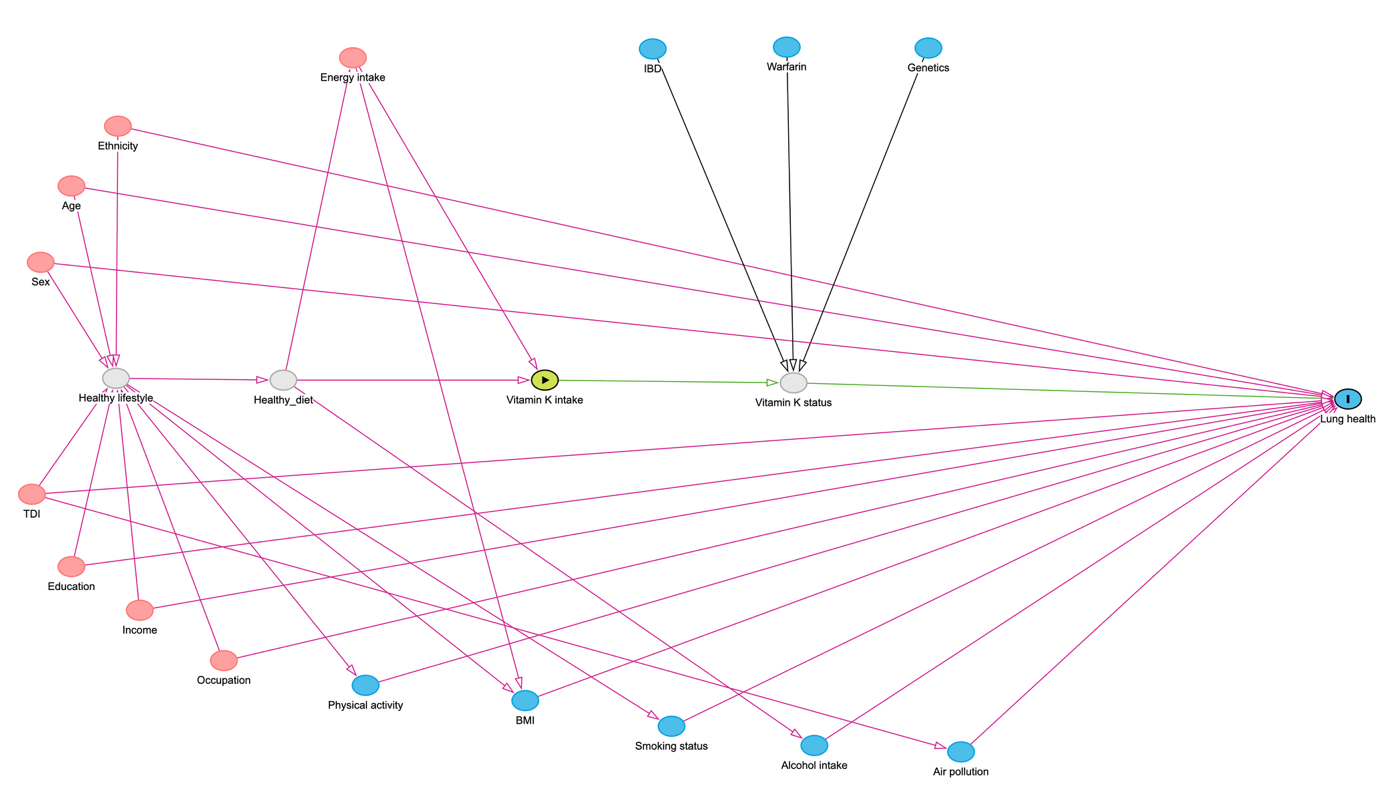


**Supplementary Figure 3.** Directed acyclic graph for the association between dietary vitamin K and lung health. BMI, body mass index; TDI, Townsend deprivation index; IBD, inflammatory bowel disease; CKD, chronic kidney disease


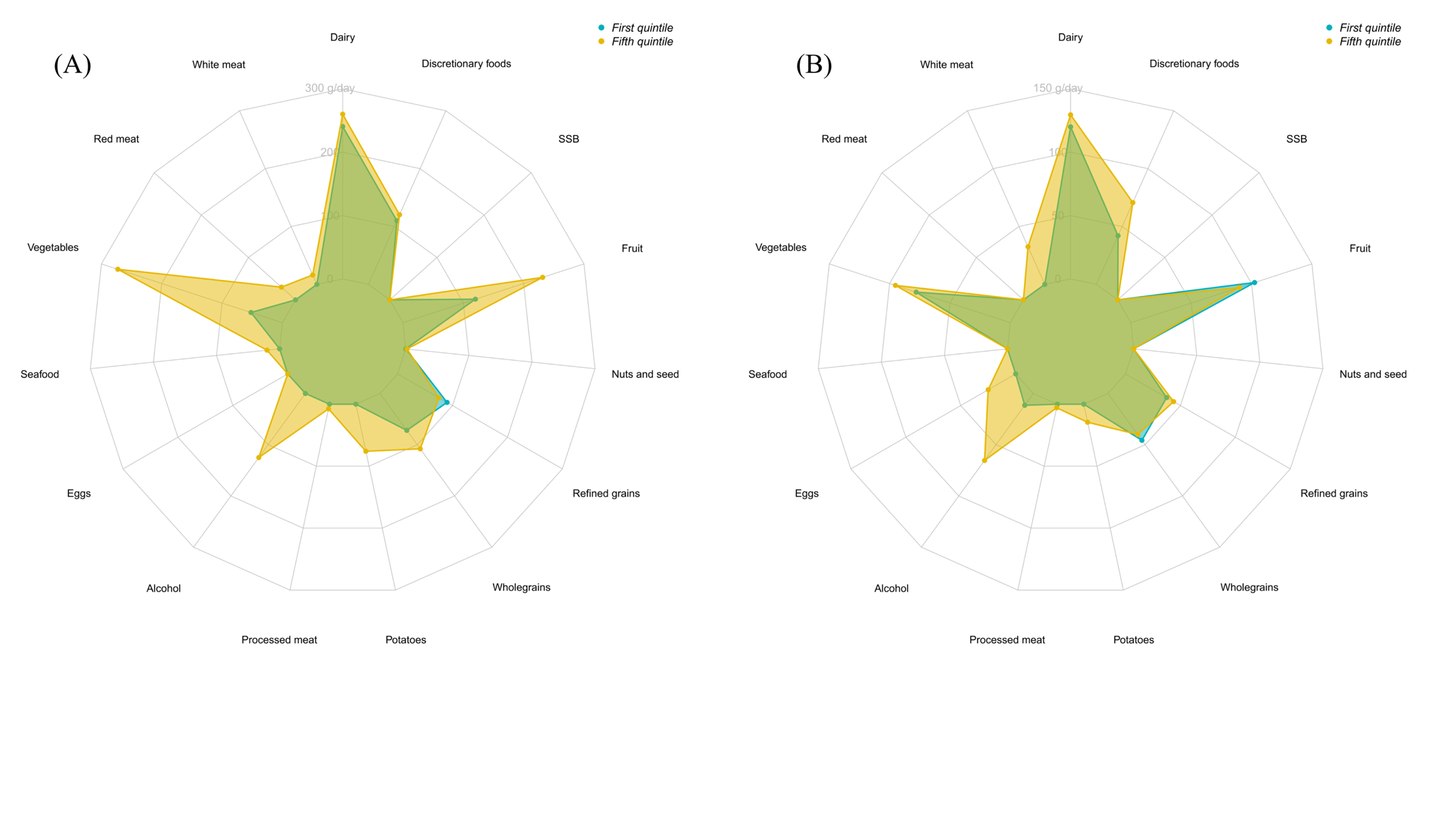
**Supplementary Figure 4.** Radar plots comparing major food group intakes between participants in the highest (quintile 5) and lowest (quintile 1) quintiles of dietary vitamin K₁ (A) and vitamin K₂ (B) in the UK Biobank study. Intakes are expressed relative to the cohort median.


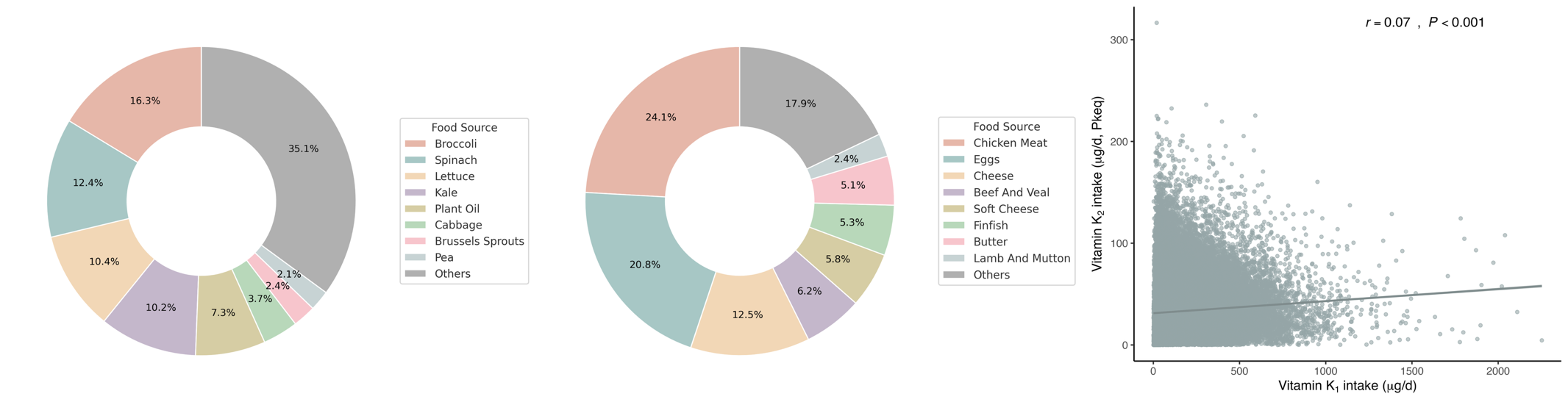


(A)

(B)

(C)

**Supplementary Figure 5.** Vitamin K intake in the UK Biobank study. (A), Composition of vitamin K_1_ intake. (B), Composition of vitamin K_2_ intake. (C), Two-sided Pearson correlation between vitamin K_1_ and K_2_ intake.

**
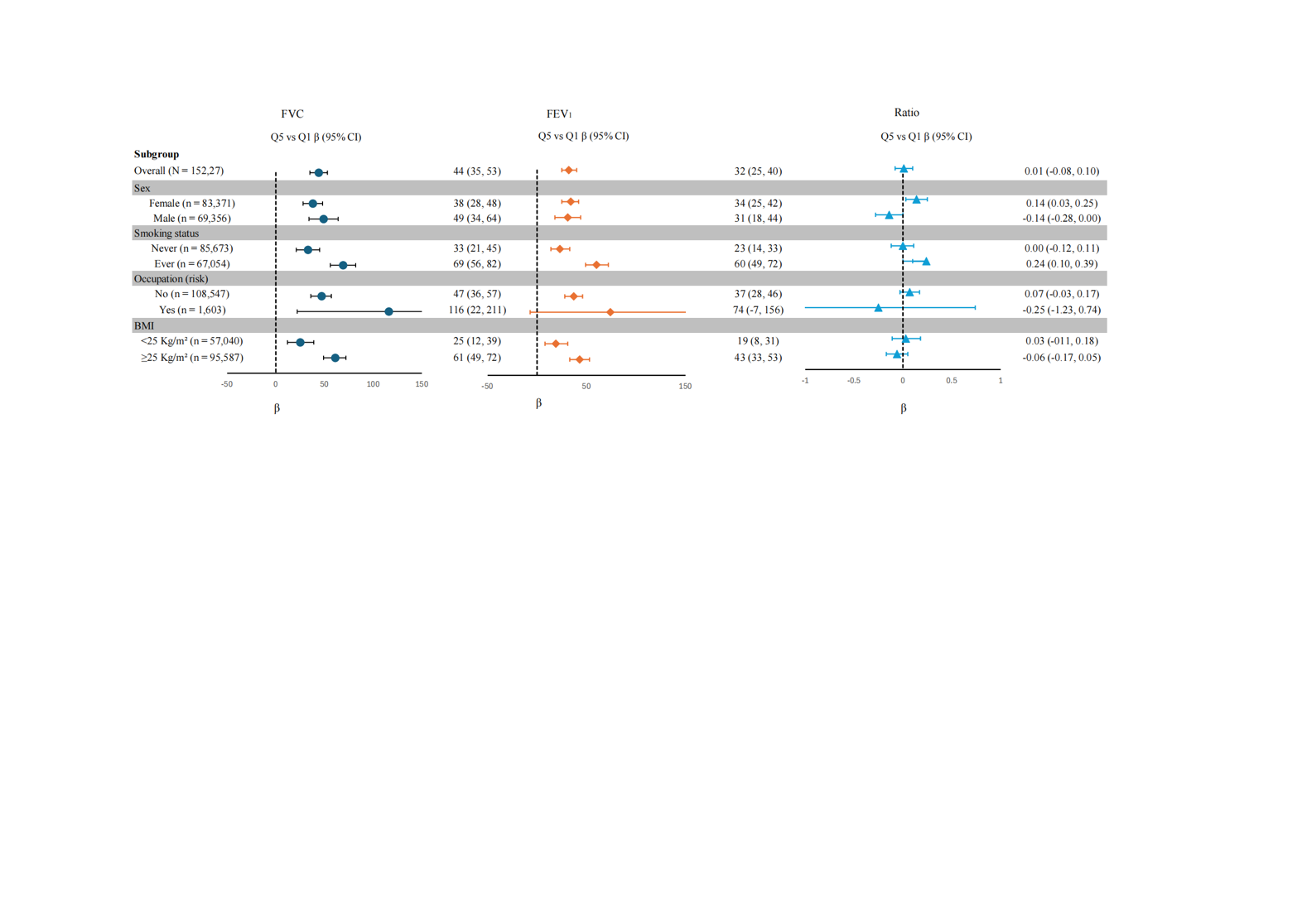
**

**Supplementary Figure 6.** Forest plots illustrating stratified associations between dietary vitamin K_1_ intake and lung function parameters—forced vital capacity (FVC, ml), forced expiratory volume in 1 second (FEV₁, ml), and the FEV₁/FVC ratio (%). Analyses were stratified by key demographic and clinical subgroups, including age, sex, smoking status, occupation, Estimated β coefficients and 95% confidence intervals (CIs) were derived from restricted cubic spline terms in linear regression models. Associations compare the median intake in the highest quintile (Q5) to that in the lowest quintile (Q1, reference). Models were comprehensively adjusted for sex, age, geographic region, height, body mass index, education, Townsend Deprivation Index, income, ethnicity, physical activity, smoking status, passive smoking, occupation, alcohol intake, number of plausible dietary recalls, dietary intakes of white meat, red and organ meats, seafood, eggs, whole grains, refined grains, potatoes, nuts and seeds, sugar-sweetened beverages, tea and coffee, and discretionary foods. P values for nonlinearity were derived from likelihood ratio tests.

**
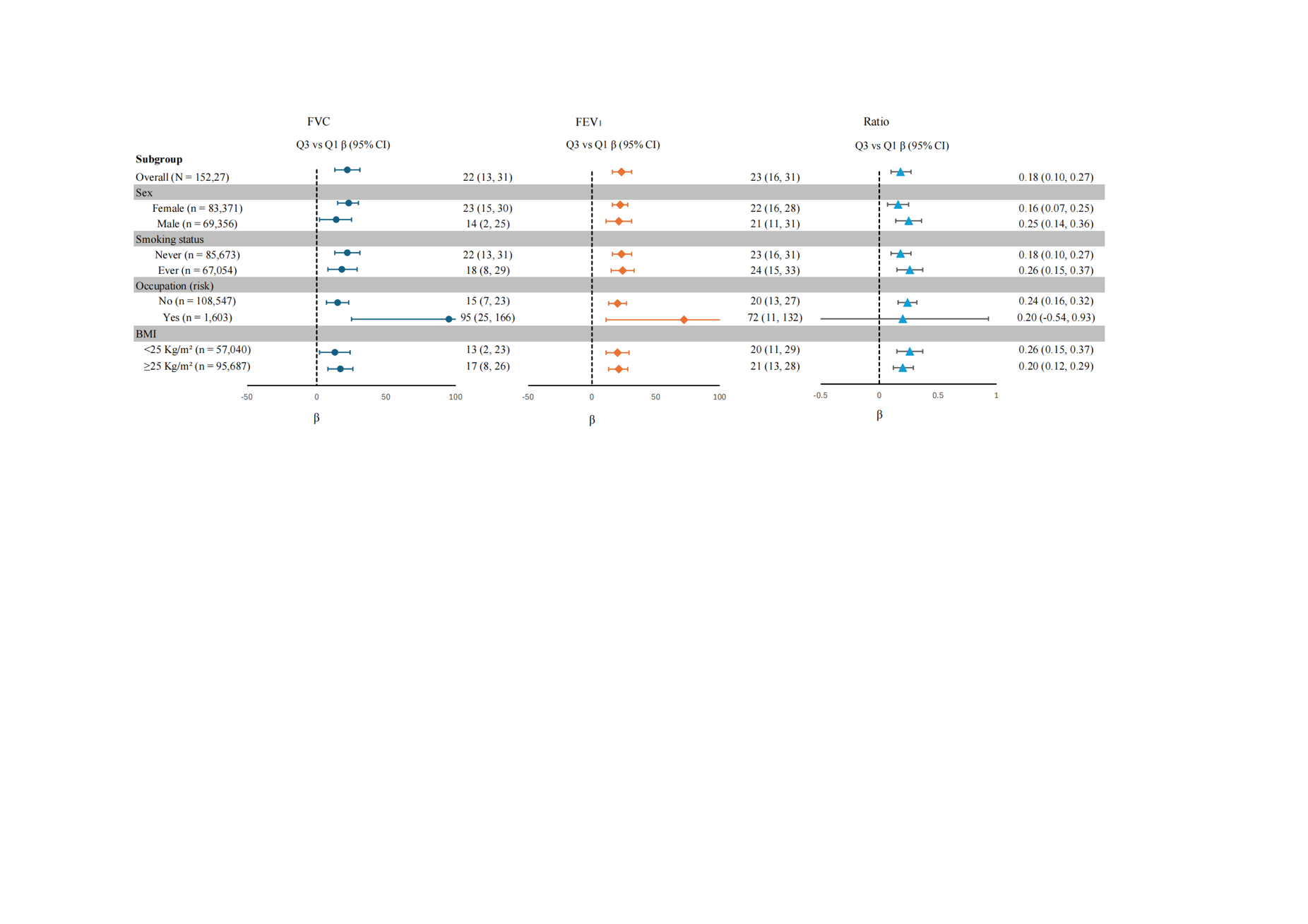
Supplementary Figure 7.** Forest plots illustrating stratified associations between dietary vitamin K_2_ intake and lung function parameters—forced vital capacity (FVC, ml), forced expiratory volume in 1 second (FEV₁, ml), and the FEV₁/FVC ratio (%). Analyses were stratified by key demographic and clinical subgroups, including age, sex, smoking status, occupation, Estimated β coefficients and 95% confidence intervals (CIs) were derived from restricted cubic spline terms in linear regression models. Associations compare the median intake in the middle quintile (Q3) to that in the lowest quintile (Q1, reference). Models were comprehensively adjusted for sex, age, geographic region, height, body mass index, education, Townsend Deprivation Index, income, ethnicity, physical activity, smoking status, passive smoking, occupation, alcohol intake, number of plausible dietary recalls, intakes of fruits, vegetables, whole grains, refined grains, potatoes, tea and coffee, and discretionary foods. P values for nonlinearity were derived from likelihood ratio tests.

| **Supplementary Table 1.** Covariate coding and categorisation information | | |
| --- | --- | --- |
| **Variables** | **Categorisation** | **UK Biobank variable description and data-field ID** |
| **Outcome** |  |  |
| COPD | No; Yes | ICD-10: COPD (J44), Data-Field 20002 |
| Asthma | No; Yes | ICD-10: COPD (J45), Data-Field 131494 |
| Forced vital capacity (FVC) | Continuous, milliliter | Data-Field 20151 |
| Forced expiratory volume in 1-second (FEV1) | Continuous, milliliter | Data-Field 20150 |
| FEV1/FVC ratio | Continuous, % |  |
| **Demographics** |  |  |
| Age | Continuous, year | Age at recruitment (ID: 21022)^*^ |
| Sex | Female; Male | Sex (ID: 31)^*^ |
| Ethnicity | White, Others | Ethnic background (ID: 21000)^*^ |
| Region | London; Wales; North-West England; North-East England; Yorkshire; West Midlands; East Midlands; South-East England; South-West England; Scotland | UK Biobank assessment centre (ID:54)^*^ |
| BMI | Underweight (<18.5 kg/m^2^); Healthy weight (18.5-24.99 kg/m^2^); Overweight (25-29.99 kg/m^2^); Obese (30-30.99 kg/m^2^) | Body mass index (ID: 21001)^§^ |
| Height | Continuous, cm | Standing height (ID:50) ^§^ |
| Waist circumference | Continuous, cm | Waist circumference (ID:48) ^§^ |
| **Socioeconomic status** |  |  |
| Education | Low: CSEs or equivalent, O levels/GCSEs or equivalent; Medium: A levels/AS levels or equivalent, NVQ or HND or HNC or equivalent; High: College or University degree, other professional qualifications eg: nursing, teaching; Unknown/Missing | Qualifications (ID: 6138)^†^ |
| Townsend deprivation index | Continuous | Townsend deprivation index (ID: 189)^*^ |
| Income | <£18,000; £18,000 – £30,999; £31,000 - £51,999; £52,000 - £100,000; >£100,000; don’t know/ prefer not to answer | Average total household income before tax (ID: 738) ^*^ |
| Occupation | High risk; Non high-risk; unknown[1] | Data-Field 22601 |
| **Diet and Lifestyle** |  |  |
| Combined smoking status | Never smokers; Former smoker ≤10 pack years; Former smoker >10 to ≤20 pack years; Former smoker >20 to ≤30 pack years; Former smoker >30 to ≤40 pack years; Former smoker >40 to ≤50 pack years; Former smoker >50 pack years; Former smoker missing packyears; Current smoker ≤5 cigarettes/day;Current smoker >5to ≤10 cigarettes/day; Current smoker >10 to ≤15 cigarettes/day; Current smoker >15 to ≤20 cigarettes/day; Current smoker >20 cigarettes/day; Current smoker missing cigarettes/day | Smoking status (ID: 20116) †; Smoking pack years (ID: 20161); Number of cigarettes currently smoked daily (current cigarette smokers only) (ID: 3456) |
| Pack years | Never smoked; 0<py≤10; 10<py≤20; 20<py≤30; 30<py≤40; 40<py≤50; 50≤py; prefer not to answer | Smoking pack years (ID: 20161)† |
| Number of cigarettes currently smoked daily as categorical variable | Never smoked; Former smoker; ≤5/day ;>5 to ≤10/day ;>10 to ≤15/day ;4>15 to ≤20/day;>20 day ; prefer not to answer | Number of cigarettes currently smoked daily (current cigarette smokers)  (ID: 3456)† |
| Passive smoker | No; Yes | Exposure to tobacco smoke at home (ID: 1269) †; Exposure to tobacco smoke outside home (ID: 1279)† |
| PM2.5 | Continuous (μg/m^3^) | Particulate matter air pollution (pm2.5); ID: 24006 |
| Physical activity | METs hr/week quintiles | Duration of walks (ID: 874)^†^; Number of days/week walked 10+ minutes (ID: 864)^†^; Duration of moderate activity (ID: 894)^†^; Number of days/week of moderate physical activity 10+ minutes (ID: 884)^†^; Duration of vigorous activity (ID: 914)^†^; Number of days/week of vigorous physical activity 10+ minutes (ID: 904)^†^. |
| Number of completed plausible recalls | 1; 2; 3; 4; 5 | Plausible recalls are only those with valid energy intakes: >800 or <4200 kilocalories/day for men and >500 or <3500 kilocalories/day for women. |
| Total dairy intake | Continuous (g/day) | 24-hr 0xford webQ; food group total, including milk (whole, semi-skimmed, and skim) yoghurt (high and low fat), cheese and cream |
| Total white meat intake | Continuous (g/day) | 24-hr 0xford webQ; food group total, including poultry and pork |
| Total red organ meat intake | Continuous (g/day) | 24-hr 0xford webQ; food group total, including beef, lamb and other red meat |
| Total processed meat | Continuous (g/day) | 24-hr 0xford webQ; food group total, including bacon, ham, sausages |
| Total seafood | Continuous (g/day) | 24-hr 0xford webQ; food group total, including white fish, shellfish and oily fish |
| Total eggs intake | Continuous (g/day) | 24-hr 0xford webQ; food group total, including eggs |
| Total vegetables intake | Continuous (g/day) | 24-hr 0xford webQ; food group total, including leafy salads, root vegetables, tomatoes, allium vegetables, legumes, peas, and other vegetables |
| Total potatoes intake | Continuous (g/day) | 24-hr 0xford webQ; food group total, including only baked and mashed potatoes (both white potato and sweet potato) |
| Total fruit intake | Continuous (g/day) | 24-hr 0xford webQ; food group total, including citrus fruits, berries, apples, pear, and dried and stewed fruit |
| Total wholegrains intake | Continuous (g/day) | 24-hr 0xford webQ; food group total, including whole grain and brown breads or rolls, oats, bran cereals, and muesli |
| Total refined grains intake | Continuous (g/day) | 24-hr 0xford webQ; food group total, white bread or rolls, crackers, sugary cereals and savoury snacks |
| Total nuts and seeds intake | Continuous (g/day) | 24-hr 0xford webQ; food group total, including slated and unsalted nuts, seeds and spreads (e.g., peanut butter) |
| Total tea and coffee intake | Continuous (g/day) | 24-hr 0xford webQ; food group total, including tea (black, green or other) and coffee (regular and decaffeinated) |
| Total alcohol intake | Continuous (g/day) | 24-hr 0xford webQ; food group total, including wine (red, white and fortified) beer and spirits |
| Total sweet soft beverages | Continuous (g/day) | 24-hr 0xford webQ; food group total, including fizzy drinks and squash |
| Total discretionary | Continuous (g/day) | 24-hr 0xford webQ; food group total, including ice-cream, chocolate sweets, pizza, samosa, fried chips biscuits. |
| **Health status** |  |  |
| Prevalent asthma | No; Yes | Any diagnosis of COPD prior to or on the date of the last valid dietary recall was identified using ICD-10 code J45. (category ID: 20002)^#^ |
| Prevalent COPD | No; Yes | Any diagnosis of COPD prior to or on the date of the last valid dietary recall was identified using ICD-10 codes J43 and J44. (category ID: 20002)# |
| Prevalent CKD | No; Yes | Non-cancer illness – self-reported (category ID: 20002)^#^ ICD-10: N18. |
| Prevalent IBD | No; Yes | Crohn’s disease (CD) was identified using ICD-10 code K50 and ICD-9 code 555, while ulcerative colitis (UC) was classified under ICD-10 code K51 and ICD-9 code 556. |
| History of lung diseases | No; Yes | Any diagnosis of lung diseases prior to or on the date of the last valid dietary recall was identified using ICD-10 code J40-43. (category ID: 20002)^#^ |
| **Medication use** |  |  |
| Warfarin use | No; Yes | Self-reported use of warfarin at baseline, extracted from data field: 20003 |
| ^*^Data collected at recruitment  ^†^Data collected via touchscreen questionnaire  ^‡^Data collected from 24-hr online Oxford WebQ dietary questionnaire  ^§^Physical measurements  ^#^Data collected via baseline interview  Abbreviations: BMI, body mass index; COPD, chronic obstructive pulmonary disease; CVD, cardiovascular disease; CKD, chronic kidney disease; IBD, inflammatory bowel disease; MET, metabolic equivalent task. | | |

Reference

1 De Matteis S, Jarvis D, Hutchings S, et al. Occupations associated with COPD risk in the large population-based UK biobank cohort study. Occup Environ Med 2016; 73: 378–384.

| **Supplementary Table 2.** Baseline dietary intakes of the study population. | | | | | |
| --- | --- | --- | --- | --- | --- |
|  | Total population N = 179,062 | Vitamin K_1_ Intake Quintiles | | Vitamin K_2_ Intake Quintiles | |
|  |  | Q1 = 35, 813 | Q5 = 35, 812 | Q1 = 35, 813 | Q5 = 35, 812 |
| Dietary energy intake, kcal | 1996 (1673, 2361) | 1793 (1467, 2162) | 2113 (1777, 2504) | 1696 (1412, 2003) | 2278 (1918, 2683) |
| Seafood, g/day | 0 (0, 46) | 0 (0, 25) | 0 (0, 50) | 0 (0, 50) | 0 (0, 37) |
| White meat, g/day | 0 (0, 49) | 0 (0, 43) | 0 (0, 65) | 0 (0, 0) | 65 (0, 130) |
| Red and organ meat, g/day | 0 (0, 60) | 0 (0, 60) | 0 (0, 80) | 0 (0, 40) | 0 (0, 60) |
| Processed meat, g/day | 0 (0, 23) | 0 (0, 23) | 0 (0, 23) | 0 (0, 12) | 6 (0, 41) |
| Dairy, g/day | 82 (33,150) | 96 (40,175) | 63 (20,127) | 75 (22,150) | 87 (33,157) |
| Eggs, g/day | 0 (0, 30) | 0 (0, 24) | 0 (0, 33) | 0 (0, 0) | 50 (0, 100) |
| Potatoes, g/day | 44 (0, 107) | 0 (0, 45) | 88 (0, 175) | 0 (0, 90) | 29 (0, 117) |
| Fruit, g/day | 191 (100, 303) | 112 (17, 216) | 243 (138, 372) | 208 (100, 322) | 180 (81, 300) |
| Vegetables, g/day | 173 (90, 276) | 45 (0, 106) | 317 (226, 444) | 157 (68, 272) | 190 (99, 305) |
| Wholegrains, g/day | 92 (41, 160) | 72 (0, 138) | 107 (49, 184) | 94 (40, 176) | 80 (36, 152) |
| Refined grains, g/day | 82 (33, 150) | 96 (40, 175) | 63 (20, 127) | 75 (21, 150) | 87 (33, 157) |
| Nuts and seeds, g/day | 0 (0, 7) | 0 (0, 2) | 0 (0, 10) | 0 (0, 7) | 0 (0, 7) |
| Tea and coffee, g/day | 770 (570, 1023) | 760 (570, 950) | 823 (570, 1077) | 760 (570, 998) | 760 (570, 1045) |
| Alcohol, g/day | 100 (0, 350) | 50 (0, 358) | 93 (0, 310) | 23 (0, 263) | 125 (0, 383) |
| Sugar-sweetened beverages, g/day | 0 (0, 125) | 0 (0, 125) | 0 (0, 83) | 0 (0, 83) | 0 (0, 125) |
| Discretionary foods, g/day | 120 (60, 204) | 120 (52, 210) | 111 (52, 197) | 84 (34, 158) | 138 (66, 231) |
| Data expressed as median (interquartile range, IQR). | | | | | |

| **Supplementary Table 3**. Hazard ratios for incident chronic obstructive pulmonary disease (COPD) by quintiles of vitamin K intake | | | | | | |
| --- | --- | --- | --- | --- | --- | --- |
|  |  | Vitamin K1 intake quintiles | | | | |
|  |  | Quintile 1 | Quintile 2 | Quintile 3 | Quintile 4 | Quintile 5 |
|  | Quintile 1 | ref | 0.81 (0.65, 1.23) | 1.02 (0.81, 1.28) | 0.80 (0.62, 1.03) | 0.72 (0.56, 0.93) |
|  | Quintile 2 | 0.81 (0.65, 1.00) | 0.92 (0.74, 1.15) | 0.78 (0.61, 0.99) | 0.86 (0.68, 1.09) | 0.86 (0.68, 1.08) |
| Vitamin K2 quintiles | Quintile 3 | 0.94 (0.76, 1.17) | 1.15 (0.94, 1.41) | 1.03 (0.83, 1.27) | 0.73 (0.58, 0.94) | 0.87 (0.69, 1.09) |
|  | Quintile 4 | 1.10 (0.90, 1.34) | 0.95 (0.77, 1.18) | 0.75 (0.59, 0.96) | 0.80 (0.64, 1.01) | 0.90 (0.73, 1.12) |
|  | Quintile 5 | 1.10 (0.90, 1.35) | 0.98 (0.79, 1.22) | 0.96 (0.78, 1.19) | 0.92 (0.74, 1.14) | 0.89 (0.72, 1.09) |
| Hazard ratios were estimated using Cox proportional hazards models (Model 2), adjusted for sex, age, geographic region, body mass index, education level, Townsend Deprivation Index, household income, ethnicity, physical activity, smoking status, exposure to passive smoking, occupation, and alcohol consumption. | | | | | | |

| **Supplementary Table 4.** Hazard ratios for incident chronic obstructive pulmonary disease (COPD) and asthma across quintiles of dietary MK-4 intake | | | | | |  |
| --- | --- | --- | --- | --- | --- | --- |
| Vitamin K intake quintiles | | | | | |  |
|  | Q1 = 35,813 | Q2 = 35,813 | Q3 = 35,812 | Q4 = 35,812 | Q5 = 35,812 | |
| MK-4, μgPKeq/d*† | 8 (5, 10) | 16 (14, 18) | 24 (22, 26) | 34 (31, 37) | 49 (43, 60) | |
| Incident COPD |  |  |  |  |  | |
| Event(n) | 573 | 592 | 604 | 645 | 721 | |
| HR (95% CI) |  |  |  |  |  | |
| Model 1 | ref. | 0.98 (0.91, 1.06) | 1.01 (0.92, 1.10) | 1.08 (0.99, 1.19) | 1.19 (1.08, 1.31) | |
| Model 2 | ref. | 1.02 (0.95, 1.11) | 1.04 (0.95, 1.14) | 1.06 (0.97, 1.16) | 1.09 (0.98, 1.20) | |
| Model 3b | ref. | 1.04 (0.96, 1.13) | 1.06(0.97, 1.17) | 1.08 (0.98, 1.19) | 1.10 (0.99, 1.22) | |
| Incident asthma |  |  |  |  |  | |
| Event(n) | 909 | 843 | 797 | 846 | 874 | |
| HR (95% CI) |  |  |  |  |  | |
| Model 1 | ref. | 0.94(0.88, 1.00) | 0.93 (0.86, 1.00) | 0.95 (0.89, 1.03) | 1.00 (0.92, 1.08) | |
| Model 2 | ref. | 0.96 (0.9, 1.02) | 0.95 (0.88, 1.02) | 0.95 (0.88, 1.02) | 0.96 (0.88, 1.04) | |
| Model 3b | ref. | 0.97 (0.9, 1.03) | 0.95 (0.88, 1.03) | 0.95 (0.88, 1.03) | 0.95 (0.88, 1.04) | |

A total of 3,135 and 4,269 participants were diagnosed with COPD and asthma, respectively, over a median follow-up period of 10.5 years. Hazard ratios (HRs) and 95% confidence intervals (CIs) were estimated using restricted cubic spline functions within Cox proportional hazards models, with pointwise estimates comparing the midpoint of each quintile to the reference midpoint of the first quintile (Q1). Model 1 adjusted for sex, age, and region; Model 2 additionally adjusted for body mass index, education, Townsend deprivation index, income, ethnicity, region, physical activity, smoking status, passive smoking, occupation, and alcohol; Model 3b adjusted for all variables in Model 2, plus fruits, vegetables, whole grains, refined grains, potatoes, tea and coffee, discretionary foods, and the number of completed plausible dietary recalls. Abbreviations: CI, confidence interval; HR, hazard ratio.

* Median (interquartile range, IQR).

† Vitamin K_2_ intakes were converted to vitamin K_1_ equivalents based on molecular weight differences to enable appropriate summation across the different vitamin K_2_ forms.

| **Supplementary Table 5.** Hazard ratios for incident chronic obstructive pulmonary disease (COPD) and asthma across quintiles of dietary MK-9 intake | | | | | |  |
| --- | --- | --- | --- | --- | --- | --- |
| Vitamin K intake quintiles | | | | | |  |
|  | Q1 = 35,813 | Q2 = 35,813 | Q3 = 35,812 | Q4 = 35,812 | Q5 = 35,812 | |
| MK-9, μgPKeq/d*† | 0.06 (0.02, 0.12) | 0.42 (0.28, 0.66) | 1.47 (1.23, 1.65) | 2.82 (2.32, 2.99) | 5.06 (4.06, 6.79) | |
| Incident COPD |  |  |  |  |  | |
| Event(n) | 788 | 654 | 556 | 599 | 538 | |
| HR (95% CI) |  |  |  |  |  | |
| Model 1 | ref. | 0.86 (0.82, 0.91) | 0.70 (0.63, 0.77) | 0.72 (0.66, 0.79) | 0.76 (0.69, 0.83) | |
| Model 2 | ref. | 0.94 (0.89, 0.99) | 0.86 (0.78, 0.95) | 0.88 (0.81, 0.97) | 0.91 (0.82, 1.00) | |
| Model 3b | ref. | 0.96 (0.91, 1.01) | 0.90 (0.81, 1.00) | 0.92 (0.83, 1.00) | 0.92 (0.84, 1.02) | |
| Incident asthma |  |  |  |  |  | |
| Event(n) | 972 | 877 | 836 | 804 | 780 | |
| HR (95% CI) |  |  |  |  |  | |
| Model 1 | ref. | 0.96 (0.92, 1.00) | 0.89 (0.81, 0.97) | 0.85 (0.79, 0.92) | 0.85 (0.78, 0.92) | |
| Model 2 | ref. | 0.99 (0.95, 1.04) | 0.96 (0.88, 1.06) | 0.93 (0.86, 1.01) | 0.92 (0.85, 1.01) | |
| Model 3b | ref. | 1.00 (0.96, 1.04) | 0.98 (0.89, 1.07) | 0.94 (0.87, 1.02) | 0.92 (0.84, 1.00) | |

A total of 3,135 and 4,269 participants were diagnosed with COPD and asthma, respectively, over a median follow-up period of 10.5 years. Hazard ratios (HRs) and 95% confidence intervals (CIs) were estimated using restricted cubic spline functions within Cox proportional hazards models, with pointwise estimates comparing the midpoint of each quintile to the reference midpoint of the first quintile (Q1). Model 1 adjusted for sex, age, and region; Model 2 additionally adjusted for body mass index, education, Townsend deprivation index, income, ethnicity, region, physical activity, smoking status, passive smoking, occupation, and alcohol; Model 3b adjusted for all variables in Model 2, plus fruits, vegetables, whole grains, refined grains, potatoes, tea and coffee, discretionary foods, and the number of completed plausible dietary recalls. Abbreviations: CI, confidence interval; HR, hazard ratio.

* Median (interquartile range, IQR).

† Vitamin K_2_ intakes were converted to vitamin K_1_ equivalents based on molecular weight differences to enable appropriate summation across the different vitamin K_2_ forms.

| **Supplementary Table 6.** Hazard ratios for incident chronic obstructive pulmonary disease (COPD) across quintiles of dietary vitamin K_1_ intake, restricted to participants with two or more completed dietary recalls. | | | | | |
| --- | --- | --- | --- | --- | --- |
| Vitamin K intake quintiles | | | | | |
|  | Q1 = 21,437 | Q2 = 21,437 | Q3 = 21,437 | Q4 = 21,437 | Q5 = 21,436 |
| Vitamin K_1_,μg/d* | 44 (34, 53) | 77 (69, 86) | 115 (105, 126) | 166 (151, 183) | 267 (230, 333) |
| Event(n) | 399 | 324 | 305 | 277 | 304 |
| HR (95% CI) |  |  |  |  |  |
| Model 1 | ref. | 0.75 (0.68, 0.82) | 0.65 (0.58, 0.73) | 0.65 (0.58, 0.73) | 0.66 (0.57, 0.75) |
| Model 2 | ref. | 0.96 (0.87, 1.05) | 0.94 (0.83, 1.05) | 0.93 (0.82, 1.05) | 0.89 (0.78, 1.03) |
| Model 3a | ref. | 0.97 (0.89, 1.07) | 0.96 (0.84, 1.08) | 0.94 (0.83, 1.07) | 0.90 (0.77, 1.04) |
| Vitamin K_2_, μg PKeq/d*† | 15 (12, 18) | 24 (22, 26) | 31 (29, 32) | 39 (36, 41) | 53 (48, 62) |
| Event(n) | 300 | 291 | 332 | 322 | 364 |
| HR (95% CI) |  |  |  |  |  |
| Model 1 | ref. | 0.92 (0.78, 1.08) | 1.03 (0.88, 1.21) | 1.01 (0.86, 1.18) | 1.18 (1.02, 1.38) |
| Model 2 | ref. | 0.92 (0.79, 1.09) | 1.03 (0.88, 1.20) | 0.96 (0.82, 1.13) | 1.04 (0.89, 1.22) |
| Model 3b | ref. | 0.91 (0.77, 1.07) | 1.01 (0.86, 1.19) | 0.95 (0.81, 1.11) | 1.01 (0.87, 1.19) |

A total of participants were diagnosed with COPD over a median follow-up of 10.5 years. Median (IQR), N = 107,184, number of events 1,609. Model 1 adjusted for sex, age, and region; Model 2 additionally adjusted for body mass index, education, Townsend deprivation index, income, ethnicity, region, physical activity, smoking status, passive smoking, occupation, and alcohol; Model 3a additionally adjusted for number of completed plausible recalls, white meat, red and organ meat, seafood, eggs, wholegrains, refined grains, potatoes, nut and seeds, sugar-sweetened beverages, tea and coffee, and discretionary foods; Model 3b adjusted for all variables in Model 2, plus fruits, vegetables, whole grains, refined grains, potatoes, tea and coffee, discretionary foods, and the number of completed plausible dietary recalls.

* Median (IQR).

† Vitamin K_2_ intakes were converted to vitamin K_1_ equivalents based on molecular weight differences to enable appropriate summation across the different vitamin K_2_ forms.

Abbreviations: CI, confidence interval; HR, hazard ratio.

| **Supplementary Table 7.** Hazard ratios for incident chronic obstructive pulmonary disease (COPD) across quintiles of dietary vitamin K1 intake, excluding participants with a diagnosis of chronic kidney disease (CKD) at baseline. | | | | | | | | | | |  |
| --- | --- | --- | --- | --- | --- | --- | --- | --- | --- | --- | --- |
| Vitamin K intake quintiles | | | | | | | | | | |  |
|  | Q1 = 35,334 | | Q2 = 35,334 | | Q3 = 35,333 | | Q4 = 35,333 | | Q5 = 35,333 | |  |
| Vitamin K_1_, μg/d* | | 34 (24, 42) | | 66 (58, 74) | | 104 (93, 116) | | 163 (145, 183) | | 287 (238, 371) | |
| Event(n) | 792 | | 636 | | 552 | | 496 | | 565 | |  |
| HR (95% CI) |  | |  | |  | |  | |  | |  |
| Model 1 | ref. | | 0.73 (0.68, 0.78) | | 0.60 (0.55, 0.65) | | 0.56 (0.52, 0.61) | | 0.58 (0.53, 0.64) | |  |
| Model 2 | ref. | | 0.95 (0.89, 1.01) | | 0.90 (0.82, 0.98) | | 0.86 (0.78, 0.93) | | 0.83 (0.75, 0.92) | |  |
| Model 3a | ref. | | 0.97 (0.90, 1.04) | | 0.93 (0.84, 1.02) | | 0.87 (0.80, 0.96) | | 0.84 (0.75, 0.94) | |  |
| Vitamin K_2_, μg PKeq/d*† | 12 (9, 15) | | 24 (22, 26) | | 31 (29, 32) | | 39 (36, 41) | | 53 (48, 62) | |  |
| Event(n) | 587 | | 543 | | 602 | | 620 | | 689 | |  |
| HR (95% CI) |  | |  | |  | |  | |  | |  |
| Model 1 | ref. | | 0.92 (0.86, 0.99) | | 0.95 (0.87, 1.03) | | 1.04 (0.95, 1.13) | | 1.15 (1.05, 1.27) | |  |
| Model 2 | ref. | | 0.99 (0.92, 1.07) | | 1.01 (0.93, 1.10) | | 1.05 (0.96, 1.15) | | 1.09 (0.99, 1.20) | |  |
| Model 3b | ref. | | 1.00 (0.93, 1.08) | | 1.03 (0.94, 1.12) | | 1.07 (0.97, 1.17) | | 1.10 (0.99, 1.21) | |  |

A total of participants were diagnosed with COPD over a median follow-up of 10.5 years. Median (IQR), N = 176,667, number of events 3,041. Model 1 adjusted for sex, age, and region; Model 2 additionally adjusted for body mass index, education, Townsend deprivation index, income, ethnicity, region, physical activity, smoking status, passive smoking, occupation, and alcohol; Model 3a additionally adjusted for number of completed plausible recalls, white meat, red and organ meat, seafood, eggs, wholegrains, refined grains, potatoes, nut and seeds, sugar-sweetened beverages, tea and coffee, and discretionary foods; Model 3b adjusted for all variables in Model 2, plus fruits, vegetables, whole grains, refined grains, potatoes, tea and coffee, discretionary foods, and the number of completed plausible dietary recalls.

* Median (IQR).

† Vitamin K_2_ intakes were converted to vitamin K_1_ equivalents based on molecular weight differences to enable appropriate summation across the different vitamin K_2_ forms.

Abbreviations: CI, confidence interval; HR, hazard ratio.

| **Supplementary Table 8**. Hazard ratios for incident chronic obstructive pulmonary disease (COPD) across quintiles of dietary vitamin K1 intake, excluding participants with a baseline diagnosis of inflammatory bowel disease (IBD). | | | | | | | | | | |  |
| --- | --- | --- | --- | --- | --- | --- | --- | --- | --- | --- | --- |
| Vitamin K intake quintiles | | | | | | | | | | |  |
|  | Q1 = 35,400 | | Q2 = 35,400 | | Q3 = 35,399 | | Q4 = 35,399 | | Q5 = 35,399 | |  |
| Vitamin K_1_, μg/d* | | 34 (24, 42) | | 66 (58, 74) | | 104 (93, 116) | | 163 (145, 183) | | 287 (239, 371) | |
| Event(n) | 795 | | 639 | | 565 | | 504 | | 565 | |  |
| HR (95% CI) |  | |  | |  | |  | |  | |  |
| Model 1 | ref. | | 0.73 (0.69, 0.78) | | 0.60 (0.55, 0.66) | | 0.57 (0.52, 0.62) | | 0.58 (0.53, 0.64) | |  |
| Model 2 | ref. | | 0.96 (0.90, 1.02) | | 0.91 (0.84, 1.00) | | 0.87 (0.79, 0.95) | | 0.84 (0.76, 0.93) | |  |
| Model 3a | ref. | | 0.98 (0.91, 1.05) | | 0.94 (0.86, 1.03) | | 0.88 (0.80, 0.97) | | 0.84 (0.75, 0.94) | |  |
| Vitamin K_2_, μg PKeq/d*† | 12 (9,15) | | 22 (20, 24) | | 30 (28, 32) | | 39 (37, 42) | | 56 (50, 68) | |  |
| Event(n) | 591 | | 555 | | 613 | | 615 | | 694 | |  |
| HR (95% CI) |  | |  | |  | |  | |  | |  |
| Model 1 | ref. | | 0.93 (0.87, 1.00) | | 0.95 (0.88, 1.03) | | 1.03 (0.94, 1.12) | | 1.14 (1.03, 1.25) | |  |
| Model 2 | ref. | | 1.00 (0.93, 1.08) | | 1.01 (0.93, 1.10) | | 1.04 (0.95, 1.13) | | 1.07 (0.97, 1.18) | |  |
| Model 3b | ref. | | 1.01 (0.94, 1.09) | | 1.03 (0.94, 1.12) | | 1.05 (0.96, 1.16) | | 1.07 (0.97, 1.19) | |  |

A total of participants were diagnosed with COPD over a median follow-up of 10.5 years. Median (IQR), N = 176,997, number of events 3068. Model 1 adjusted for sex, age, and region; Model 2 additionally adjusted for body mass index, education, Townsend deprivation index, income, ethnicity, region, physical activity, smoking status, passive smoking, occupation, and alcohol; Model 3a additionally adjusted for number of completed plausible recalls, white meat, red and organ meat, seafood, eggs, wholegrains, refined grains, potatoes, nut and seeds, sugar-sweetened beverages, tea and coffee, and discretionary foods; Model 3b adjusted for all variables in Model 2, plus fruits, vegetables, whole grains, refined grains, potatoes, tea and coffee, discretionary foods, and the number of completed plausible dietary recalls.

* Median (IQR).

† Vitamin K_2_ intakes were converted to vitamin K_1_ equivalents based on molecular weight differences to enable appropriate summation across the different vitamin K_2_ forms.

Abbreviations: CI, confidence interval; HR, hazard ratio.

| **Supplementary Table 9.** Hazard ratios for incident chronic obstructive pulmonary disease (COPD) across quintiles of dietary vitamin K1 intake, excluding participants diagnosed with COPD within the first two years of follow-up. | | | | | |  |
| --- | --- | --- | --- | --- | --- | --- |
| Vitamin K intake quintiles | | | | | |  |
|  | Q1 = 35,736 | Q2 = 35,735 | Q3 = 35,735 | Q4 = 35,735 | Q5 = 35,735 | |
| Vitamin K_1_, μg/d* | 34 (24, 42) | 66 (58, 74) | 104 (93, 116) | 163 (145, 183) | 287 (238, 371) | |
| Event(n) | 720 | 570 | 504 | 461 | 494 | |
| HR (95% CI) |  |  |  |  |  | |
| Model 1 | ref. | 0.73 (0.68, 0.78) | 0.60 (0.54, 0.65) | 0.56 (0.52, 0.62) | 0.58 (0.52, 0.64) | |
| Model 2 | ref. | 0.95 (0.89, 1.02) | 0.90 (0.82, 0.99) | 0.85 (0.78, 0.94) | 0.83 (0.74, 0.92) | |
| Model 3a | ref. | 0.98 (0.91, 1.05) | 0.94 (0.85, 1.04) | 0.88 (0.80, 0.98) | 0.84 (0.75, 0.94) | |
| Vitamin K_2_, μg PKeq/d*† | 12 (9, 15) | 22 (20, 24) | 30 (28, 32) | 39 (37, 42) | 56 (50, 68) | |
| Event(n) | 544 | 495 | 544 | 553 | 613 | |
| HR (95% CI) |  |  |  |  |  | |
| Model 1 | ref. | 0.92 (0.85, 0.99) | 0.93 (0.85, 1.01) | 1.00 (0.91, 1.09) | 1.11 (1.00, 1.22) | |
| Model 2 | ref. | 0.99 (0.91, 1.06) | 0.99 (0.91, 1.08) | 1.01 (0.92, 1.11) | 1.05 (0.95, 1.16) | |
| Model 3b | ref. | 1.00 (0.92, 1.08) | 1.01 (0.92, 1.11) | 1.03 (0.94, 1.14) | 1.06 (0.95, 1.17) | |

A total of participants were diagnosed with COPD over a median follow-up of 10.5 years. Median (IQR), N =178,676, number of events 2,749. Model 1 adjusted for sex, age, and region; Model 2 additionally adjusted for body mass index, education, Townsend deprivation index, income, ethnicity, region, physical activity, smoking status, passive smoking, occupation, and alcohol; Model 3a additionally adjusted for number of completed plausible recalls, white meat, red and organ meat, seafood, eggs, wholegrains, refined grains, potatoes, nut and seeds, sugar-sweetened beverages, tea and coffee, and discretionary foods; Model 3b adjusted for all variables in Model 2, plus fruits, vegetables, whole grains, refined grains, potatoes, tea and coffee, discretionary foods, and the number of completed plausible dietary recalls.

* Median (IQR).

† Vitamin K_2_ intakes were converted to vitamin K_1_ equivalents based on molecular weight differences to enable appropriate summation across the different vitamin K_2_ forms.

Abbreviations: CI, confidence interval; HR, hazard ratio.

| **Supplementary Table 10.** Hazard ratios for incident chronic obstructive pulmonary disease (COPD) and asthma by quintiles of vitamin K intake with additionally adjusted for PM2.5 and history of lung diseases. | | | | | |
| --- | --- | --- | --- | --- | --- |
| N = 179,062 | Vitamin K intake quintiles | | | | |
|  | Q1 = 35,813 | Q2 = 35,813 | Q3 = 35,812 | Q4 = 35,812 | Q5 = 35,812 |
| Vitamin K_1_, (μg/d) * | 34 (24, 42) | 66 (58, 74) | 104 (93, 116) | 163 (145, 183) | 287 (238, 371) |
| Incident COPD | | | | | |
| Cases/n | 817/35,813 | 652/35,813 | 575/35,812 | 513/35,812 | 578/35,812 |
| HR (95% CI) |  |  |  |  |  |
| Model 1 | ref. | 0.73 (0.69, 0.78) | 0.60 (0.55, 0.65) | 0.56 (0.52, 0.61) | 0.58 (0.52, 0.64) |
| Model 2 | ref. | 0.96 (0.90, 1.02) | 0.91 (0.84, 1.00) | 0.87 (0.80, 0.95) | 0.84 (0.76, 0.94) |
| Model 3a | ref. | 0.98 (0.92, 1.05) | 0.95 (0.86, 1.04) | 0.90 (0.82, 0.99) | 0.86 (0.77, 0.96) |
| Incident asthma | | | | | |
| Cases/n | 914/35,813 | 867/35,813 | 815/35,812 | 804/35,812 | 869/35,812 |
| HR (95% CI) |  |  |  |  |  |
| Model 1 | ref. | 0.91 (0.86, 0.97) | 0.86 (0.80, 0.93) | 0.84 (0.78, 0.91) | 0.87 (0.80, 0.94) |
| Model 2 | ref. | 0.98 (0.92, 1.04) | 0.96 (0.88, 1.04) | 0.95 (0.88, 1.02) | 0.96 (0.88, 1.05) |
| Model 3a | ref. | 1.00 (0.94, 1.06) | 1.00 (0.91, 1.09) | 0.99 (0.91, 1.08) | 1.00 (0.91, 1.10) |
| Vitamin K_2_, (μg PKeq/d) *† | 12 (9, 15) | 22 (20, 24) | 30 (28, 32) | 39 (37, 42) | 56 (50, 68) |
| Incident COPD |  |  |  |  |  |
| Cases/n | 606/35,813 | 562/35,813 | 624/35,812 | 634/35,812 | 709/35,812 |
| HR (95% CI) |  |  |  |  |  |
| Model 1 | ref. | 0.93 (0.87, 1.00) | 0.95 (0.87, 1.03) | 1.03 (0.94, 1.12) | 1.14 (1.04, 1.25) |
| Model 2 | ref. | 1.00 (0.93, 1.07) | 1.02 (0.93, 1.11) | 1.05 (0.96, 1.14) | 1.08 (0.98, 1.19) |
| Model 3b | ref. | 1.01 (0.94, 1.09) | 1.03 (0.95, 1.13) | 1.06 (0.97, 1.17) | 1.09 (0.98, 1.20) |
| Incident asthma |  |  |  |  |  |
| Cases/n | 916/35,813 | 835/35,813 | 790/35,812 | 880/35,812 | 848/35,812 |
| HR (95% CI) |  |  |  |  |  |
| Model 1 | ref. | 0.93 (0.88, 0.98) | 0.92 (0.86, 0.98) | 0.95 (0.88, 1.02) | 0.99 (0.91, 1.07) |
| Model 2 | ref. | 0.97 (0.91, 1.03) | 0.95 (0.89, 1.02) | 0.95 (0.88, 1.03) | 0.96 (0.88, 1.04) |
| Model 3b | ref. | 0.97 (0.91, 1.03) | 0.96 (0.89, 1.03) | 0.95 (0.88, 1.03) | 0.95 (0.87, 1.03) |
| A total of 3,135 and 4,269 participants were diagnosed with COPD and asthma, respectively, over a median follow-up period of 10.5 years. Hazard ratios (HRs) and 95% confidence intervals (CIs) were estimated using restricted cubic spline functions within Cox proportional hazards models, with pointwise estimates comparing the midpoint of each quintile to the reference midpoint of the first quintile (Q1). Model 1 adjusted for sex, age, and region; Model 2 additionally adjusted for body mass index, education, Townsend deprivation index, income, ethnicity, region, physical activity, smoking status, passive smoking, occupation, and alcohol, PM2.5, history of lung diseases; Model 3a additionally adjusted for number of completed plausible recalls, white meat, red and organ meat, seafood, eggs, wholegrains, refined grains, potatoes, nut and seeds, sugar-sweetened beverages, tea and coffee, and discretionary foods; Model 3b adjusted for all variables in Model 2, plus fruits, vegetables, whole grains, refined grains, potatoes, tea and coffee, discretionary foods, and the number of completed plausible dietary recalls. Abbreviations: CI, confidence interval; HR, hazard ratio.  * Median (interquartile range, IQR).  † Vitamin K_2_ intakes were converted to vitamin K_1_ equivalents based on molecular weight differences to enable appropriate summation across the different vitamin K_2_ forms. | | | | | |

| **Supplementary Table 11.** Cross-sectional associations between dietary vitamin K intake and lung function parameters β (95% CI), restricted to participants with at least one dietary recall completed in the same cycle of lung function measurement at baseline. | | | | | | |
| --- | --- | --- | --- | --- | --- | --- |
|  | Vitamin K intake quintiles | | | | |  |
| N = 49,381 | Q1 = 9,877 | Q2 = 9,876 | Q3 = 9,876 | Q4 = 9,876 | Q5 = 9,876 |  |
| Vitamin K1, (μg/d)* | 29 (21, 36) | 55 (49, 62) | 85 (77, 93) | 123 (112, 135) | 203 (172, 254) |  |
| FVC, ml |  |  |  |  |  |  |
| Model 1 | ref. | 92 (81, 104) | 152 (136, 168) | 170 (155, 185) | 165 (149, 182) |  |
| Model 2 | ref. | 30 (20, 41) | 51 (37, 65) | 59 (45, 72) | 55 (41, 70) |  |
| Model 3a | ref. | 29 (19, 40) | 50 (35, 64) | 57 (43, 71) | 54 (39, 70) |  |
| FEV1, ml |  | 77 (67, 86) | 125 (112, 138) | 135 (123, 148) | 126 (113, 140) |  |
| Model 1 | ref. | 28 (20, 37) | 46 (34, 59) | 50 (39, 62) | 44 (31, 57) |  |
| Model 2 | ref. | 28 (20, 37) | 46 (34, 59) | 51 (39, 63) | 45 (32, 58) |  |
| Model 3a | ref. | 92 (81, 104) | 152 (136, 168) | 170 (155, 185) | 165 (149, 182) |  |
| FEV1/ FVC, ratio (%) |  |  |  |  |  |  |
| Model 1 | ref. | 0.23 (0.13, 0.34) | 0.34 (0.19, 0.48) | 0.25 (0.12, 0.39) | 0.11 (-0.04, 0.26) |  |
| Model 2 | ref. | 0.17 (0.06, 0.27) | 0.24 (0.10, 0.39) | 0.19 (0.05, 0.32) | 0.09 (-0.06, 0.24) |  |
| Model 3a | ref. | 0.18 (0.08, 0.29) | 0.26 (0.12, 0.41) | 0.21 (0.07, 0.36) | 0.12 (-0.03, 0.28) |  |
| Vitamin K2, (μg PKeq/d) *† | 12 (8,14) | 21 (19, 23) | 29 (27, 32) | 39 (37, 42) | 55 (49, 67) |  |
| FVC, ml |  |  |  |  |  |  |
| Model 1 | ref. | 21 (19, 23) | 29 (27, 32) | 39 (37, 42) | 55 (49, 67) |  |
| Model 2 | ref. | 50 (38, 62) | 60 (47, 74) | 46 (32, 61) | 22 (6, 38) |  |
| Model 3b | ref. | 44 (33, 56) | 60 (46, 73) | 58 (44, 72) | 50 (34, 65) |  |
| FEV1, ml |  | 46 (34, 58) | 61 (47, 74) | 58 (43, 72) | 48 (32, 64) |  |
| Model 1 | ref. | 47 (38, 57) | 58 (47, 69) | 47 (35, 59) | 26 (13, 39) |  |
| Model 2 | ref. | 40 (31, 50) | 54 (43, 65) | 51 (39, 63) | 41 (28, 54) |  |
| Model 3b | ref. | 43 (34, 53) | 57 (46, 68) | 54 (42, 66) | 44 (30, 57) |  |
| FEV1/ FVC, ratio (%) |  |  |  |  |  |  |
| Model 1 | ref. | 0.28 (0.17, 0.39) | 0.37 (0.25, 0.49) | 0.35 (0.22, 0.48) | 0.27 (0.12, 0.41) |  |
| Model 2 | ref. | 0.21 (0.11, 0.32) | 0.26 (0.14, 0.38) | 0.21 (0.08, 0.34) | 0.11 (-0.03, 0.25) |  |
| Model 3b | ref. | 0.25 (0.14, 0.36) | 0.32 (0.20, 0.44) | 0.28 (0.15, 0.41) | 0.19 (0.04, 0.33) |  |

Estimates (β) and 95% CIs were obtained from restricted cubic splines within linear regression models comparing the median exposure intakes in quintiles 2–5 to the median exposure intakes in quintile 1. Model 1 adjusted for sex, age, and region; Model 2 additionally adjusted for height, body mass index, education, Townsend deprivation index, income, ethnicity, region, physical activity, smoking status, passive smoking, occupation, and alcohol; Model 3a additionally adjusted for number of completed plausible recalls, white meat, red and organ meat, seafood, eggs, wholegrains, refined grains, potatoes, nut and seeds, sugar-sweetened beverages, tea and coffee, and Discretionary foods. Model 3b adjusted for all variables in Model 2, plus fruits, vegetables, whole grains, refined grains, potatoes, tea and coffee, discretionary foods, and the number of completed plausible dietary recalls. 52 159 participants had missing data for predicted FEV1 as their spirometry did not meet European Respiratory Society/American Thoracic Society Criteria.

† Vitamin K2 intakes were converted to vitamin K1 equivalents based on molecular weight differences to enable appropriate summation across the different Vitamin K2 forms. Abbreviations: CI, confidence interval; HR, hazard ratio. FEV1, forced expiratory volume in 1 s; FVC, forced vital capacity.

| **Supplementary Table 12.** Baseline characteristics of the study population | | | | | |
| --- | --- | --- | --- | --- | --- |
|  | Total population  N = 52,159 | Vitamin K_1_ intake quintiles | | Vitamin K_2_ intake quintiles | |
|  |  | Q1  n = 10,432 | Q5  n = 10,431 | Q1  n = 10,432 | Q5  n = 10,431 |
| Vitamin K_1_, μg/d | 101 (55, 184) | 31 (22, 39) | 295 (242, 380) | 82 (42, 163) | 112 (60, 2034) |
| Vitamin K_2_, μg PKeq/d ^†^ | 29 (19, 43) | 25 (15, 40) | 32 (20, 45) | 11 (8, 14) | 57 (51, 70) |
| Sex (female) | 29759 (57.1) | 5309 (50.9) | 6563 (62.9) | 6496 (62.3) | 5272 (50.5) |
| Age (y) | 60 (52, 65) | 57 (49, 63) | 61 (53, 66) | 59 (51, 64) | 58 (51, 65) |
| Ethnicity |  |  |  |  |  |
| White | 43060 (82.6) | 8066 (77.3) | 8615 (82.6) | 8038 (77.1) | 8250 (79.1) |
| Others | 9097 (17.4) | 2366 (22.7) | 1816 (17.4) | 2394 (22.9) | 2181 (20.9) |
| Region |  |  |  |  |  |
| London | 13266 (25.4) | 2795 (26.8) | 2825 (27.1) | 3036 (29.1) | 2750 (26.4) |
| Wales | 1534 ( 2.9) | 294 ( 2.8) | 273 ( 2.6) | 259 ( 2.5) | 317 ( 3.0) |
| North-West England | 5820 (11.2) | 1265 (12.1) | 1052 (10.1) | 1155 (11.1) | 1237 (11.9) |
| North-East England | 4803 ( 9.2) | 966 ( 9.3) | 985 ( 9.4) | 890 ( 8.5) | 926 ( 8.9) |
| Yorkshire | 7623 (14.6) | 1594 (15.3) | 1477 (14.2) | 1479 (14.2) | 1468 (14.1) |
| West Midlands | 5656 (10.8) | 1335 (12.8) | 1026 ( 9.8) | 1206 (11.6) | 1170 (11.2) |
| East Midlands | 3154 ( 6.0) | 505 ( 4.8) | 686 ( 6.6) | 562 ( 5.4) | 594 ( 5.7) |
| South-East England | 4021 ( 7.7) | 573 ( 5.5) | 864 ( 8.3) | 682 ( 6.5) | 769 ( 7.4) |
| South-West England | 4189 ( 8.0) | 699 ( 6.7) | 908 ( 8.7) | 797 ( 7.6) | 797 ( 7.6) |
| Scotland | 2093 ( 4.0) | 406 ( 3.9) | 335 ( 3.2) | 366 ( 3.5) | 403 ( 3.9) |
| Education |  |  |  |  |  |
| Low | 8081 (15.5) | 1978 (19.0) | 1524 (14.6) | 1670 (16.0) | 1589 (15.2) |
| Medium | 9031 (17.3) | 2132 (20.4) | 1645 (15.8) | 1781 (17.1) | 1917 (18.4) |
| High | 29779 (57.1) | 4867 (46.7) | 6233 (59.8) | 5719 (54.8) | 5936 (56.9) |
| Unknown | 5268 (10.1) | 1455 (13.9) | 1029 ( 9.9) | 1262 (12.1) | 989 ( 9.5) |
| Deprivation index | -2.02 (-3.57, 0.64) | -1.56 (-3.33, 1.39) | -2.05 (-3.60, 0.60) | -1.69 (-3.41, 1.07) | -1.86 (-3.49, 0.90) |
| Income |  |  |  |  |  |
| <£18,000 | 8436 (16.2) | 1962 (18.8) | 1822 (17.5) | 1853 (17.8) | 1649 (15.8) |
| £18,000–£30,999 | 11799 (22.6) | 2327 (22.3) | 2394 (23.0) | 2420 (23.2) | 2272 (21.8) |
| £31,000–£51,999 | 12669 (24.3) | 2452 (23.5) | 2408 (23.1) | 2382 (22.8) | 2529 (24.2) |
| £52,000–£100,000 | 10121 (19.4) | 1824 (17.5) | 1921 (18.4) | 1832 (17.6) | 2164 (20.7) |
| >£100,000 | 2704 ( 5.2) | 433 ( 4.2) | 559 ( 5.4) | 458 ( 4.4) | 597 ( 5.7) |
| Unknown | 6430 (12.3) | 1434 (13.7) | 1327 (12.7) | 1487 (14.3) | 1220 (11.7) |
| BMI (kg/m^2^) |  |  |  |  |  |
| Normal (18.5-24.9) | 19224 (36.9) | 3228 (30.9) | 4124 (39.5) | 4111 (39.4) | 3169 (30.4) |
| Underweight (<18.5) | 454 ( 0.9) | 62 ( 0.6) | 115 ( 1.1) | 118 ( 1.1) | 58 ( 0.6) |
| Overweight (25.0, 29.9) | 20919 (40.1) | 4357 (41.8) | 4013 (38.5) | 4077 (39.1) | 4359 (41.8) |
| Obese (30.0, 39.9) | 10593 (20.3) | 2513 (24.1) | 2003 (19.2) | 1971 (18.9) | 2551 (24.5) |
| Morbidly obese (≥40.0) | 969 ( 1.9) | 272 ( 2.6) | 176 ( 1.7) | 155 ( 1.5) | 294 ( 2.8) |
| Physical activity, MET* (h/week) | 18.40 (7.78, 38.37) | 16.05 (5.90, 36.05) | 21.32 (9.45, 43.05) | 18.62 (8.03, 38.85) | 18.58 (7.67, 39.71) |
| Occupation |  |  |  |  |  |
| Non-high risk | 36503 (70.0) | 7561 (72.5) | 7013 (67.2) | 7406 (71.0) | 7410 (71.0) |
| High-risk | 708 ( 1.4) | 222 ( 2.1) | 114 ( 1.1) | 175 ( 1.7) | 145 ( 1.4) |
| Unknown | 14948 (28.7) | 2649 (25.4) | 3304 (31.7) | 2851 (27.3) | 2876 (27.6) |
| Smoking status |  |  |  |  |  |
| Never smokers | 31061 (59.6) | 5936 (56.9) | 6124 (58.7) | 6464 (62.0) | 5992 (57.4) |
| Former smoker | 16943 (32.5) | 3240 (31.1) | 3554 (34.1) | 3135 (30.1) | 3504 (33.6) |
| Current smoker | 3937 ( 7.5) | 1199 (11.5) | 704 ( 6.7) | 773 ( 7.4) | 893 ( 8.6) |
| Prefer not to answer | 218 ( 0.4) | 57 ( 0.5) | 49 ( 0.5) | 60 ( 0.6) | 42 ( 0.4) |
| Pack years of smoking | |  |  |  |  |
| Never smoked | 31061 (59.6) | 5936 (56.9) | 6124 (58.7) | 6464 (62.0) | 5992 (57.4) |
| >0 to ≤10 pack years | 4063 ( 7.8) | 768 ( 7.4) | 902 ( 8.6) | 768 ( 7.4) | 813 ( 7.8) |
| >10 to ≤20 pack years | 3798 ( 7.3) | 827 ( 7.9) | 734 ( 7.0) | 711 ( 6.8) | 807 ( 7.7) |
| >20 to ≤30 pack years | 2371 ( 4.5) | 586 ( 5.6) | 452 ( 4.3) | 427 ( 4.1) | 499 ( 4.8) |
| >30 to ≤40 pack years | 1517 ( 2.9) | 407 ( 3.9) | 293 ( 2.8) | 263 ( 2.5) | 342 ( 3.3) |
| >40 to ≤50 pack years | 788 ( 1.5) | 194 ( 1.9) | 146 ( 1.4) | 139 ( 1.3) | 187 ( 1.8) |
| >50 pack years | 872 ( 1.7) | 215 ( 2.1) | 174 ( 1.7) | 131 ( 1.3) | 232 ( 2.2) |
| Prefer not to answer | 7689 (14.7) | 1499 (14.4) | 1606 (15.4) | 1529 (14.7) | 1559 (14.9) |
| Current smoking intensity | | |  |  |  |
| Never smoked | 31061 (59.6) | 5936 (56.9) | 6124 (58.7) | 6464 (62.0) | 5992 (57.4) |
| Former smoker | 16943 (32.5) | 3240 (31.1) | 3554 (34.1) | 3135 (30.1) | 3504 (33.6) |
| ≤5/day | 360 ( 0.7) | 99 ( 0.9) | 73 ( 0.7) | 70 ( 0.7) | 85 ( 0.8) |
| >5 to ≤10/day | 705 ( 1.4) | 233 ( 2.2) | 122 ( 1.2) | 171 ( 1.6) | 146 ( 1.4) |
| >10 to ≤15/day | 562 ( 1.1) | 192 ( 1.8) | 96 ( 0.9) | 112 ( 1.1) | 116 ( 1.1) |
| >15 to ≤20/day | 545 ( 1.0) | 197 ( 1.9) | 83 ( 0.8) | 98 ( 0.9) | 121 ( 1.2) |
| >20 day | 276 ( 0.5) | 101 ( 1.0) | 44 ( 0.4) | 38 ( 0.4) | 70 ( 0.7) |
| Prefer not to answer | 1707 ( 3.3) | 434 ( 4.2) | 335 ( 3.2) | 344 ( 3.3) | 397 ( 3.8) |
| Passive smoker | 8988 (17.2) | 2039 (19.5) | 1726 (16.5) | 1803 (17.3) | 2033 (19.5) |

Data expressed as median (IQR) or n (%).

† Vitamin K_2_ intakes were converted to vitamin K_1_ equivalents based on molecular weight differences to enable appropriate summation across the different vitamin K_2_ forms.

* MET, metabolic equivalent of task.
